# Supplementary material for: Water-stable boroxine structure with dynamic covalent bonds
Source: Nat Commun. 2024 Feb 8;15:1207. doi: 10.1038/s41467-024-45464-z (PMC10853236; doi:10.1038/s41467-024-45464-z)
Supplement: Supplementary file 1 — Supplementary information [file 41467_2024_45464_MOESM1_ESM.pdf]

Supplementary information

Water-stable boroxine structure with dynamic covalent bonds

Xiaopei Li[1,2,#], Yongjie Zhang[2,#], Zhenqiang Shi[1], Dongdong Wang[1], Hang Yang[1], Yahui Zhang[1], Haijuan Qin[3], Wenqi Lu[1], Junjun Chen[1], Yan Li[1] and Guangyan Qing[1,4,*]

[1]CAS Key Laboratory of Separation Science for Analytical Chemistry, Dalian Institute of Chemical Physics, Chinese Academy of Sciences, Dalian, P. R. China.

[2]Instrumental Analysis Center, School of Textile and Material Engineering, Dalian Polytechnic University, Dalian, P. R. China.

[3]Research Centre of Modern Analytical Technology, Tianjin University of Science & Technology, Tianjin, P. R. China

[4]College of Chemistry and Chemical Engineering, Wuhan Textile University, Wuhan, P. R. China.

*Corresponding authors: qinggy@dicp.ac.cn

#X.L. and Y.Z. contributed equally to this work

Table of contents

Supplementary Figures 1–33..2

Supplementary Tables 1–11..38

Supplementary references.. 52

Supplementary Figures 1–33

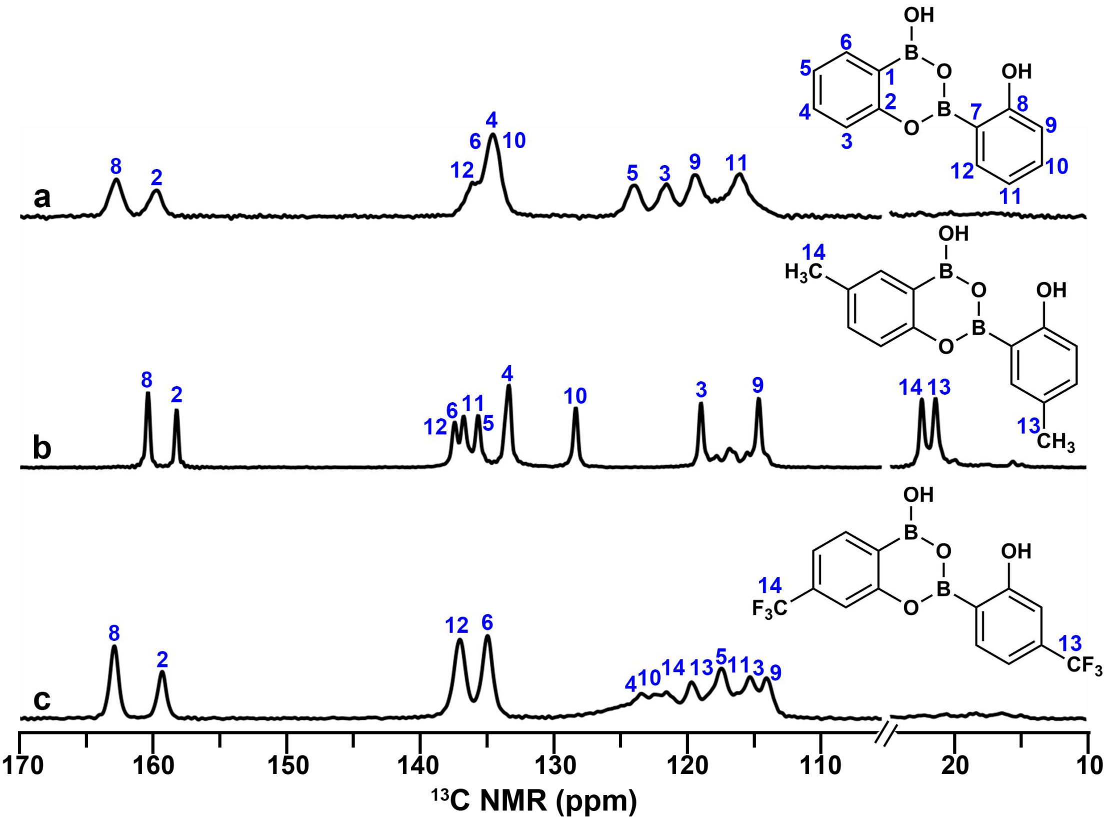

Supplementary Fig. 1 a–**c** Solid-state ^{13}C NMR spectrum of HO-PBA chemical (**a**, Aladdin Reagent, Shanghai, Product No. H101964), CH_3-HO-PBA chemical (**b**, Bide Pharmatech, Shanghai, Product No. BD217886), and CF_3-HO-PBA chemical (**c**, Leyan, Shanghai, Product No. 1201389).

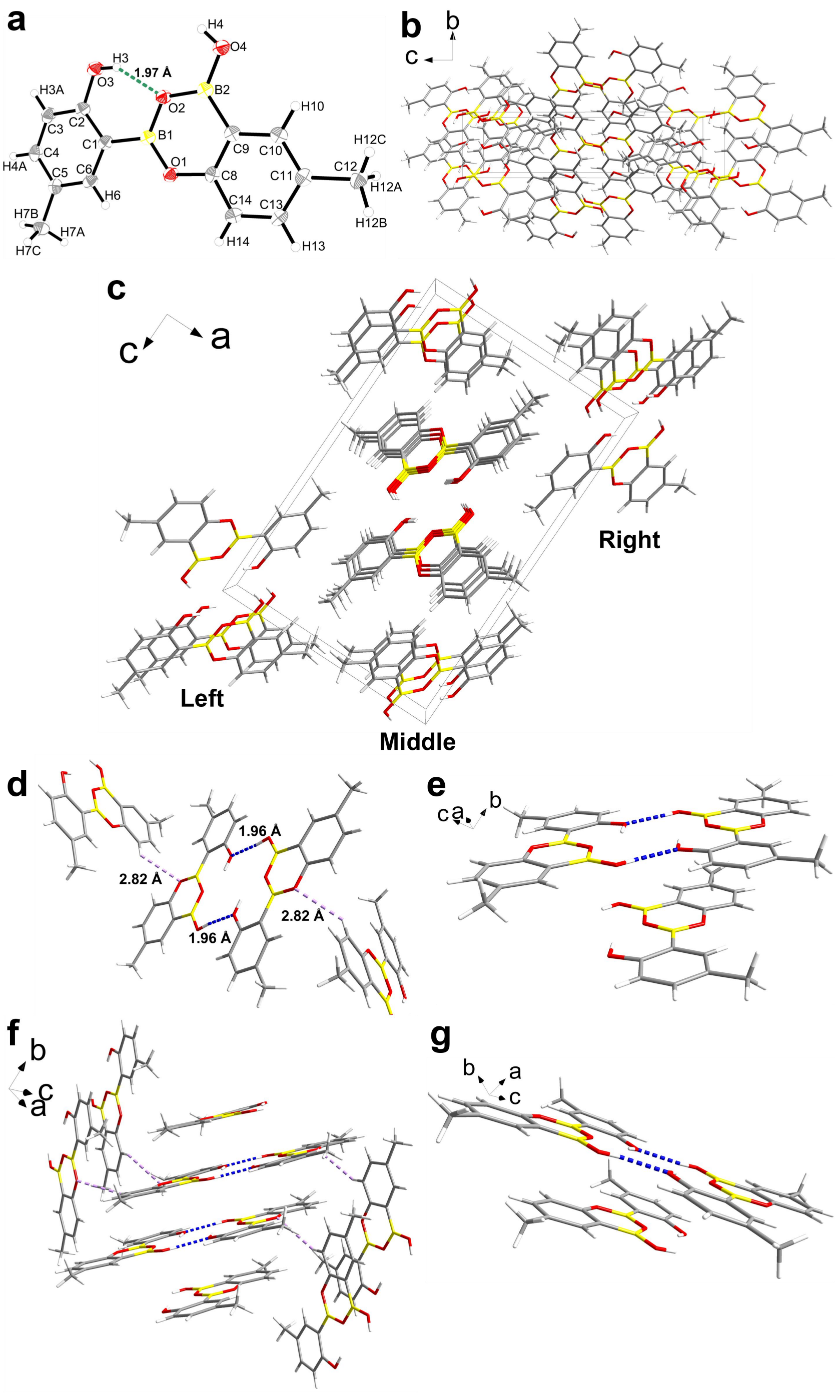

Supplementary Fig. 2 Crystal structure of CH_3-HO-PBA dimer. a Crystal structure of CH_3-HO-PBA dimer with thermal ellipsoids (CCDC 2280485). The dashed green line represents an intramolecular O–H…O hydrogen bond. **b,c** Packing mode of CH_3-HO-PBA dimers viewed along the *a* axis (**b**) and *b* axis (**c**). **d** Packing interactions of CH_3-HO-PBA dimers. The dashed blue and purple lines represent intramolecular O–H…O and C–H…O hydrogen bonds, respectively. **e-g** Packing mode of CH_3-HO-PBA dimers in the left part (**e**), middle part (**f**), and right part (**g**) of (**c**).

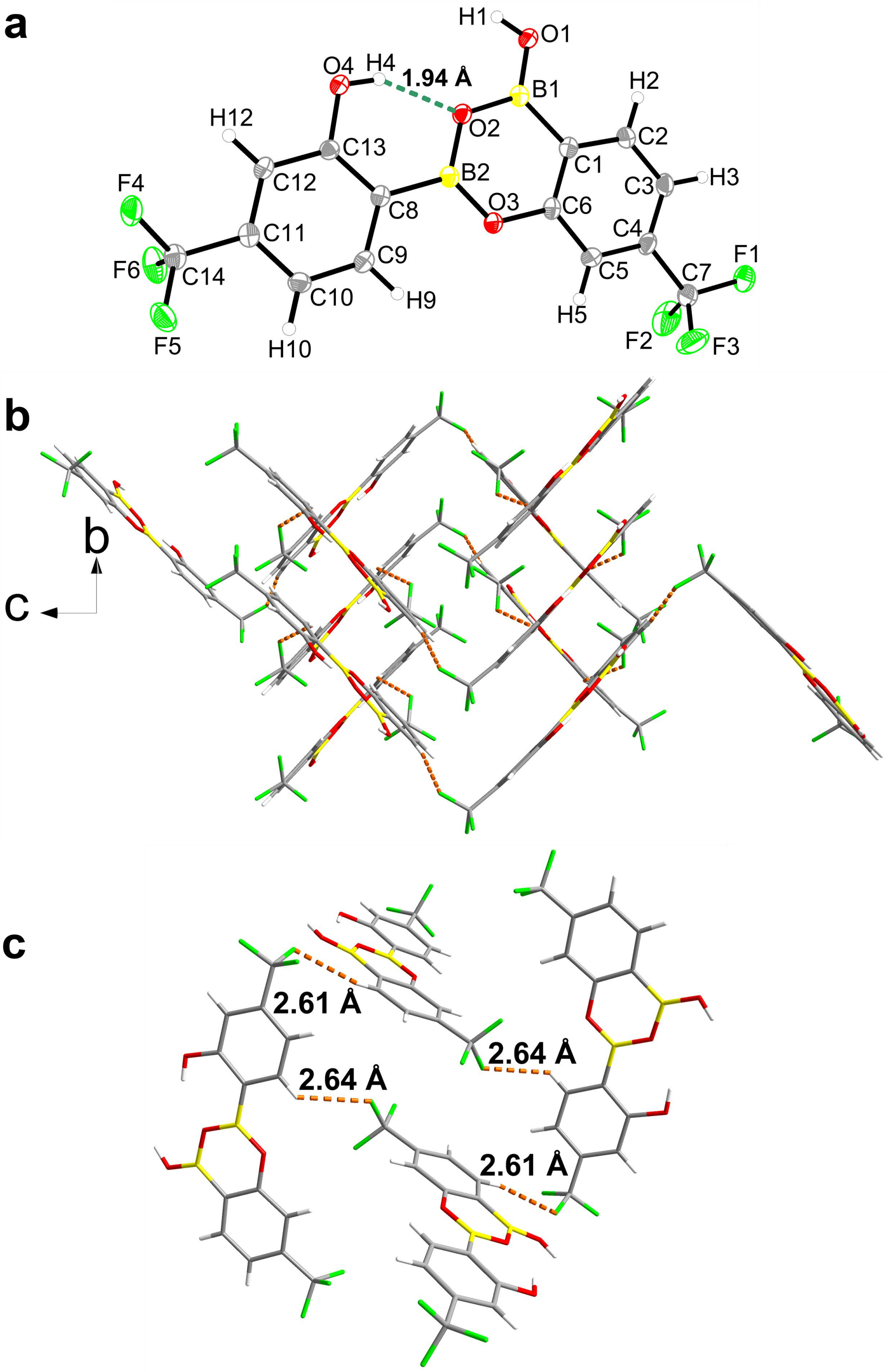

Supplementary Fig. 3 Crystal structure of CF_3-HO-PBA dimer. **a** Crystal structure of CF_3-HO-PBA dimer with thermal ellipsoids (CCDC 2280486). The dashed green line represents an intramolecular O–H…O hydrogen bond. **b** Packing mode of CF_3-HO-PBA dimers viewed along the *a* axis. **c** Packing interactions of CF_3-HO-PBA dimers. The dashed orange line represents an intramolecular C–H…F hydrogen bond.

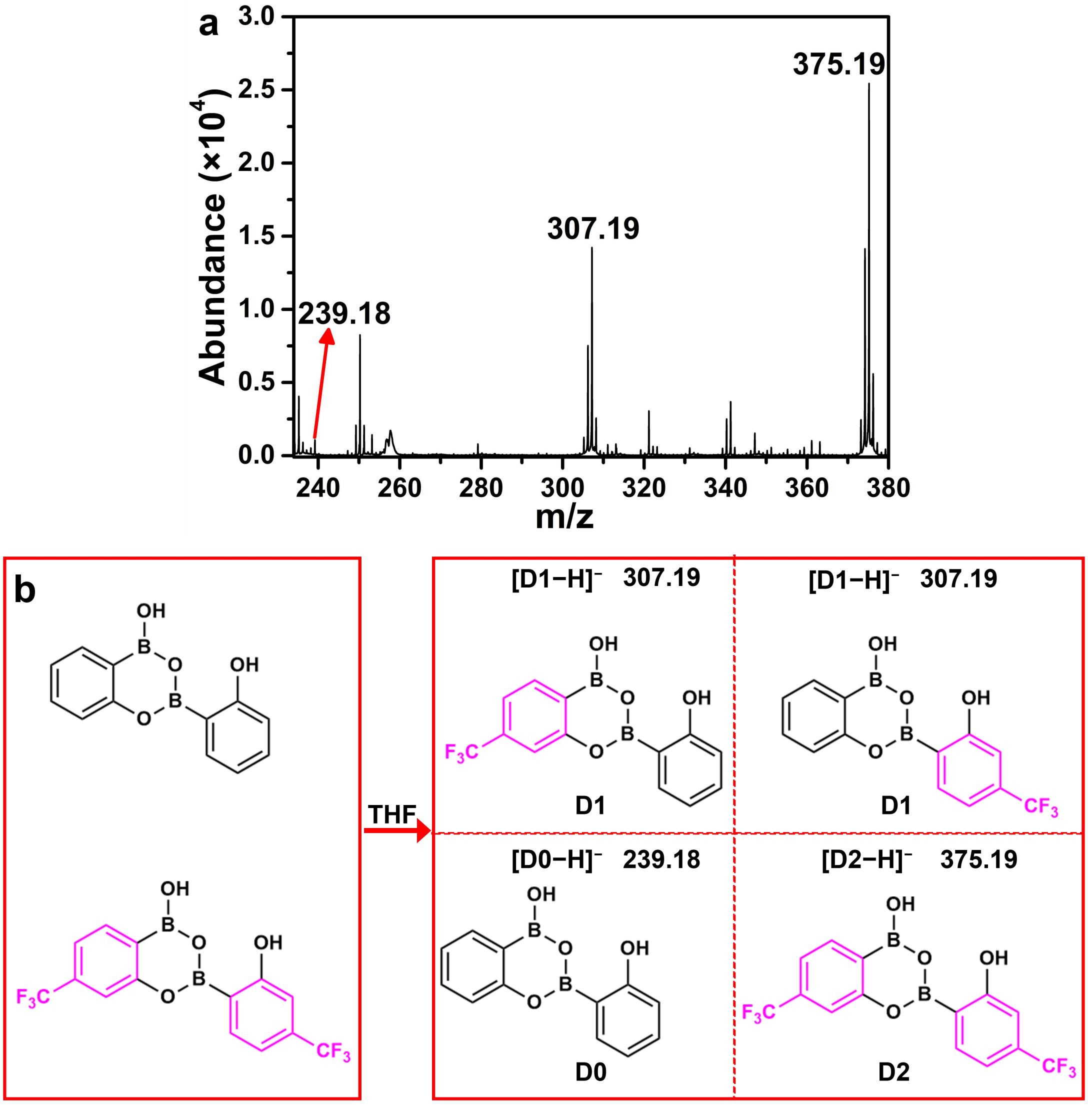

Supplementary Fig. 4 Exchange reaction between HO-PBA dimers and CF_3-HO-PBA dimers. **a** MALDI-TOF mass spectrum of products from the mixture of HO-PBA dimers and CF_3-HO-PBA dimers, acquired in a negative mode. **b** Illustration of the dynamic exchange between HO-PBA dimer and CF_3-HO-PBA dimer.

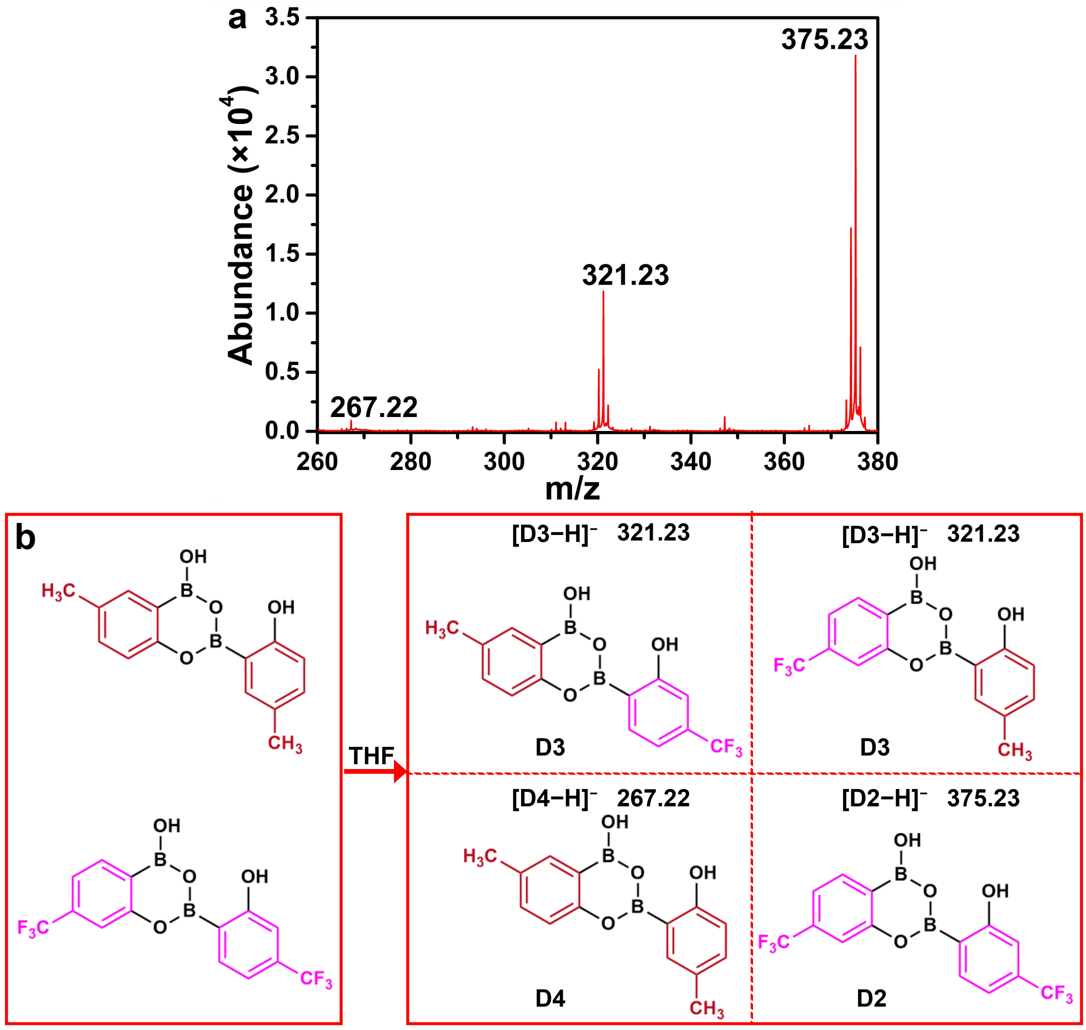

Supplementary Fig. 5 Exchange reaction between CH_3-HO-PBA dimers and CF_3-HO-PBA dimers. **a** MALDI-TOF mass spectrum of products from the mixture of CH_3-HO-PBA dimers and CF_3-HO-PBA dimers, acquired in a negative mode. **b** Illustration of the dynamic exchange between CH_3-HO-PBA dimer and CF_3-HO-PBA dimer.

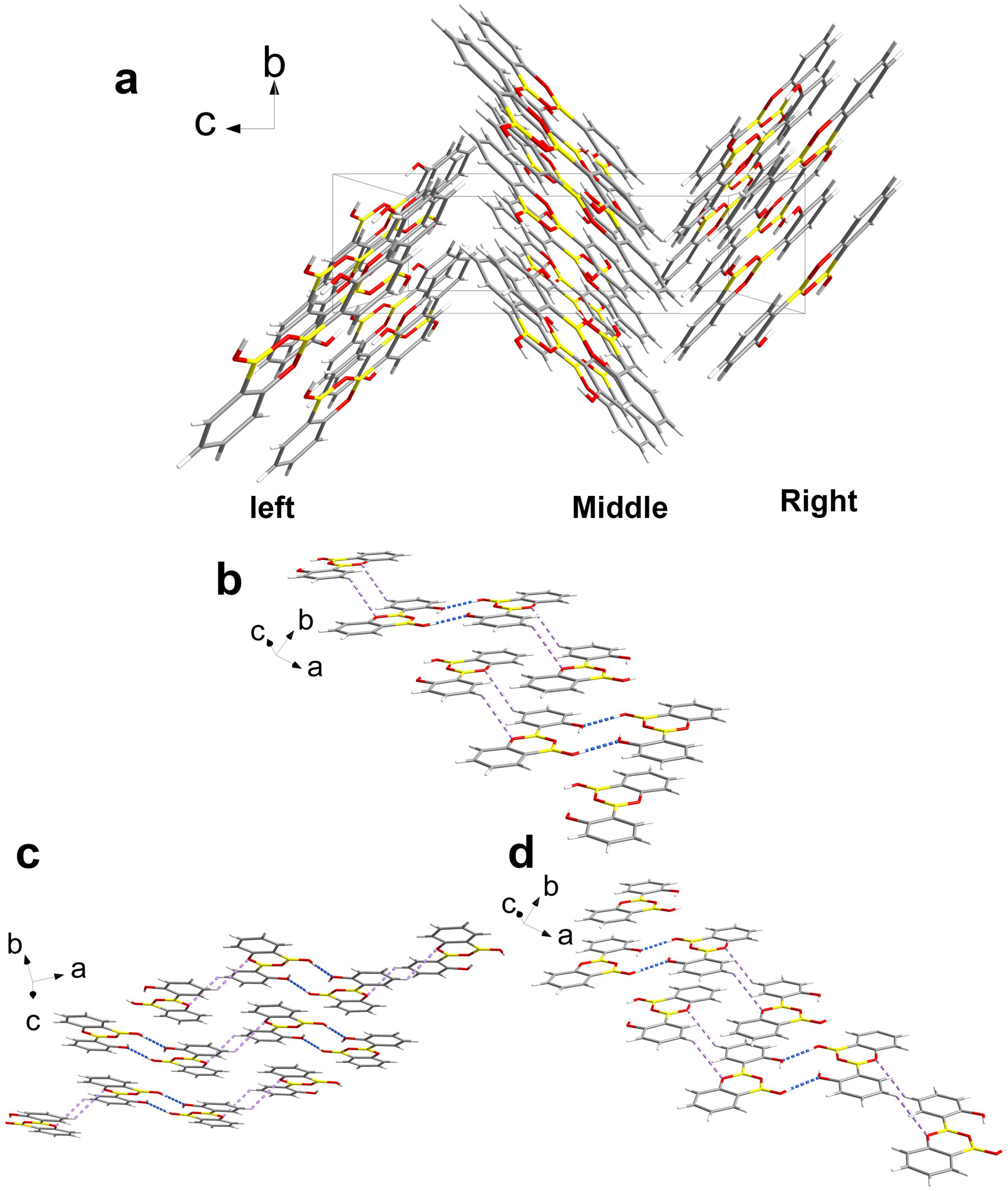

Supplementary Fig. 6 Crystal structure of HO-PBA dimer. a Packing mode of HO-PBA dimers viewed along the *a* axis. **b-d** Packing mode of HO-PBA dimers in the left part (**b**), the middle part (**c**) and right part (**d**) of (**a**).

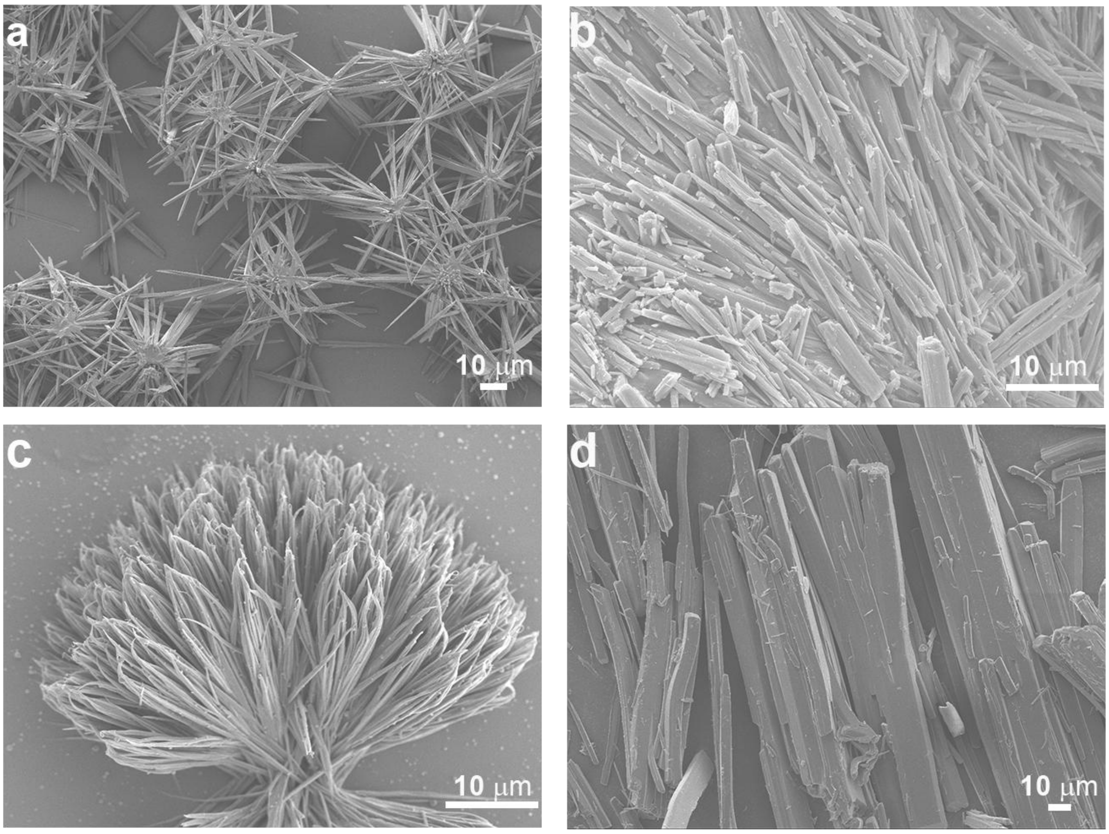

Supplementary Fig. 7 SEM images of CH_3-HO-PBA dimer and CF_3-HO-PBA dimer. a,**c** SEM images displaying assembled morphology of CH_3-HO-PBA dimer (**a**) and CF_3-HO-PBA dimer (**c**), where the sample was prepared by dropping one droplet of CH_3-HO-PBA or CF_3-HO-PBA solution on a silicon wafer. **b**,**d** SEM images of the solid CH_3-HO-PBA dimer (**b**) and CF_3-HO-PBA dimer (**d**) sample.

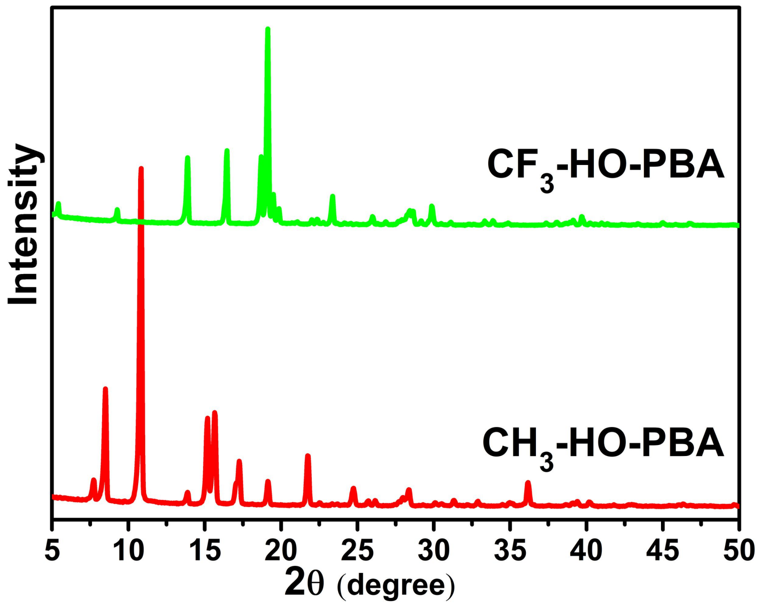

Supplementary Fig. 8 XRD patterns of the solid CH_3-HO-PBA dimer and CF_3-HO-PBA dimer sample.

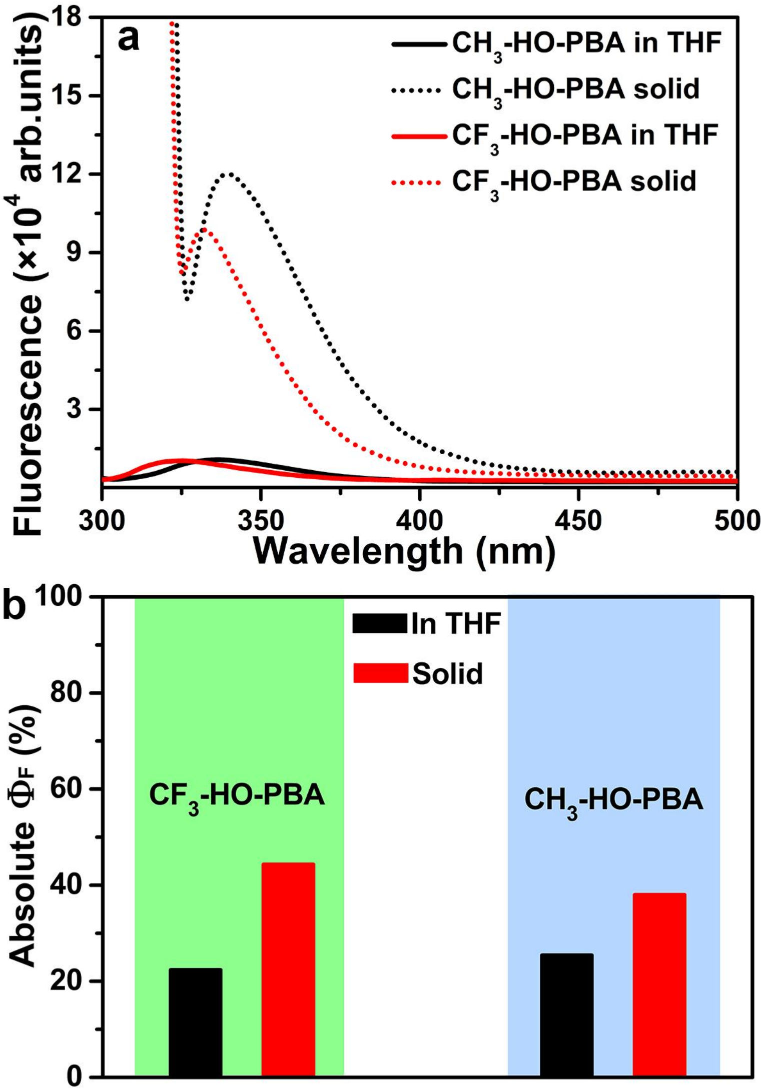

Supplementary Fig. 9 Luminescent behavior of CH_3-HO-PBA dimer and CF_3-HO-PBA dimer. a Fluorescence spectra of CH_3-HO-PBA dimer (2×10^{-5} mol·L^{-1}, λ_{ex}=298 nm) and CF_3-HO-PBA dimer (2×10^{-5} mol·L^{-1}, λ_{ex}=295 nm) in THF solution, as well as the solid CH_3-HO-PBA dimer (λ_{ex}=320 nm) and CF_3-HO-PBA dimer (λ_{ex}=318 nm) sample. **b** The absolute quantum yield (Φ_F) of CH_3-HO-PBA dimer and CF_3-HO-PBA dimer in THF solution and in the solid state.

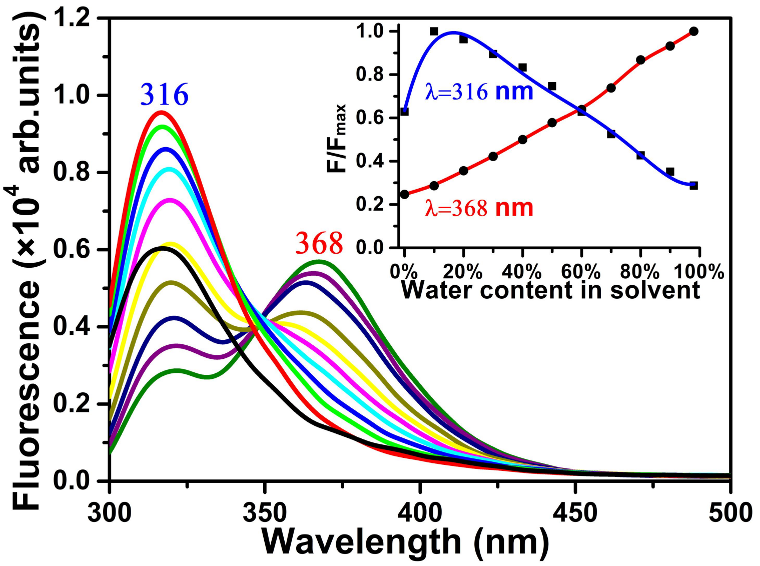

Supplementary Fig. 10 Fluorescent spectra and intensity changes (at 316 nm and 368 nm, inset) of HO-PBA dimer (2×10^{-5} mol·L^{-1}) in methanol–water mixtures with different amounts of water (volume%), λ_{ex}=288 nm.

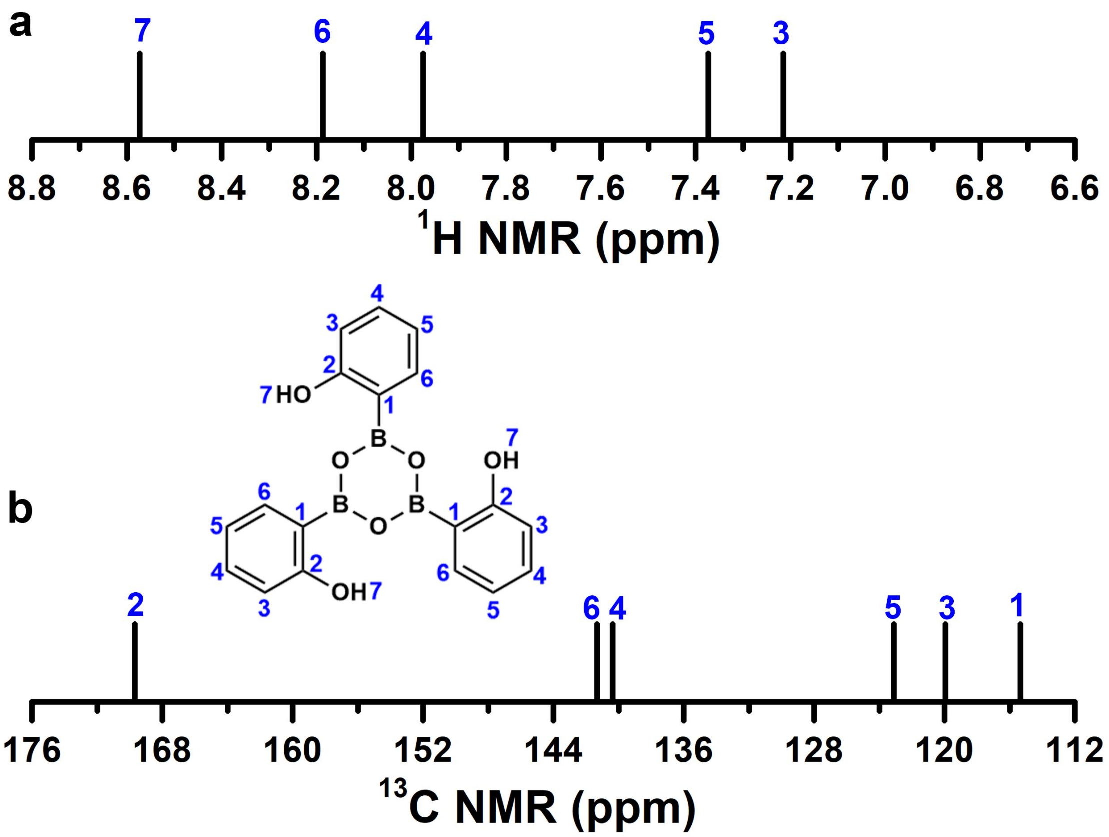

Supplementary Fig. 11 a,b Calculated ^{1}H (**a**) and ^{13}C (**b**) NMR spectra of HO-PBA trimer in water by Gaussian at the B3LYP/6-31G* level.

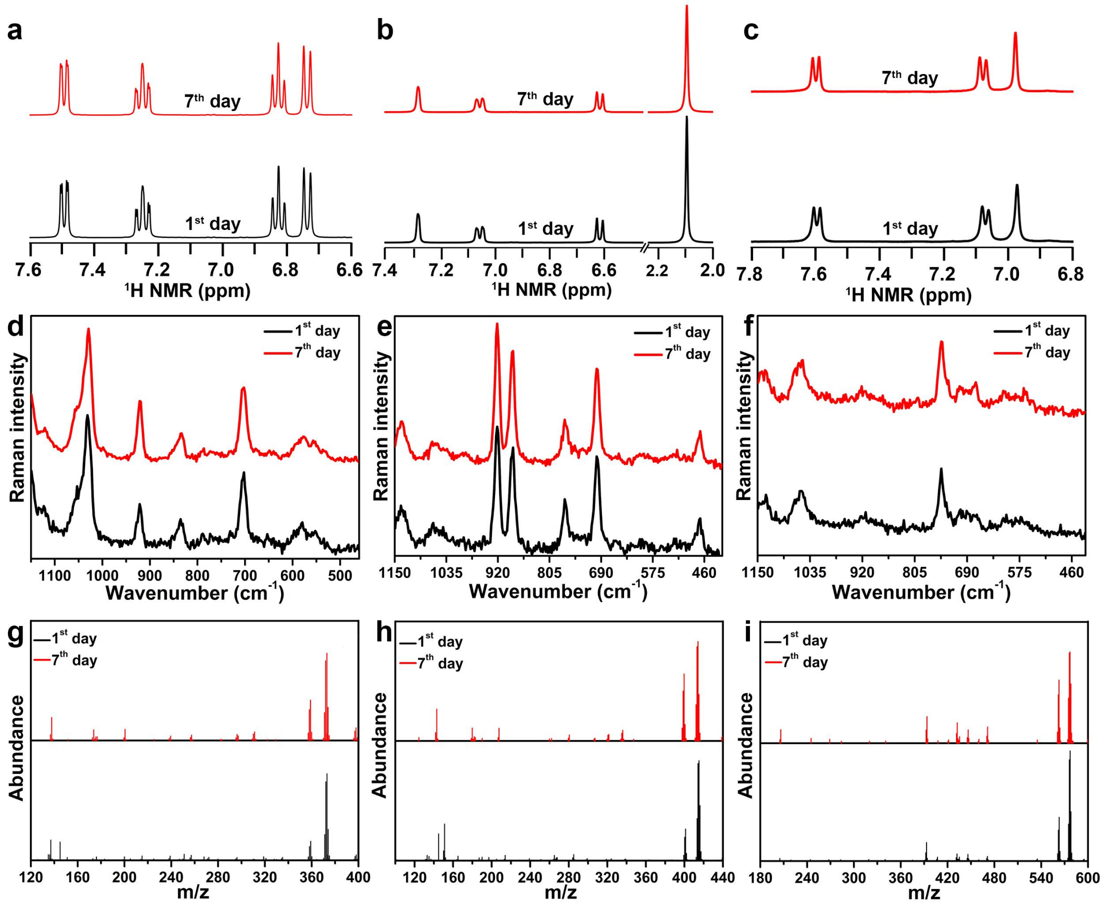

Supplementary Fig. 12 Stability of the boroxine structure with time. a–c Comparison of ^{1}H NMR spectra of HO-PBA trimer (**a**), CH_3-HO-PBA trimer (**b**), or CF_3-HO-PBA trimer (**c**) in a D_2O–DMSO–d_6 (2:1, v/v) solution on the 1st and 7th day. **d–f** Comparison of UV–Raman spectra of HO-PBA trimer (**d**), CH_3-HO-PBA trimer (**e**), or CF_3-HO-PBA trimer (**f**) in an ACN–water (1:2, v/v) solution on the 1st and 7th day. **g–i** Comparison of ESI-Q-TOF mass spectra of HO-PBA trimer (**g**), CH_3-HO-PBA trimer (**h**), or CF_3-HO-PBA trimer (**i**) in a methanol–water (5:1, v/v) solution on the 1st and 7th day, acquired in a negative mode.

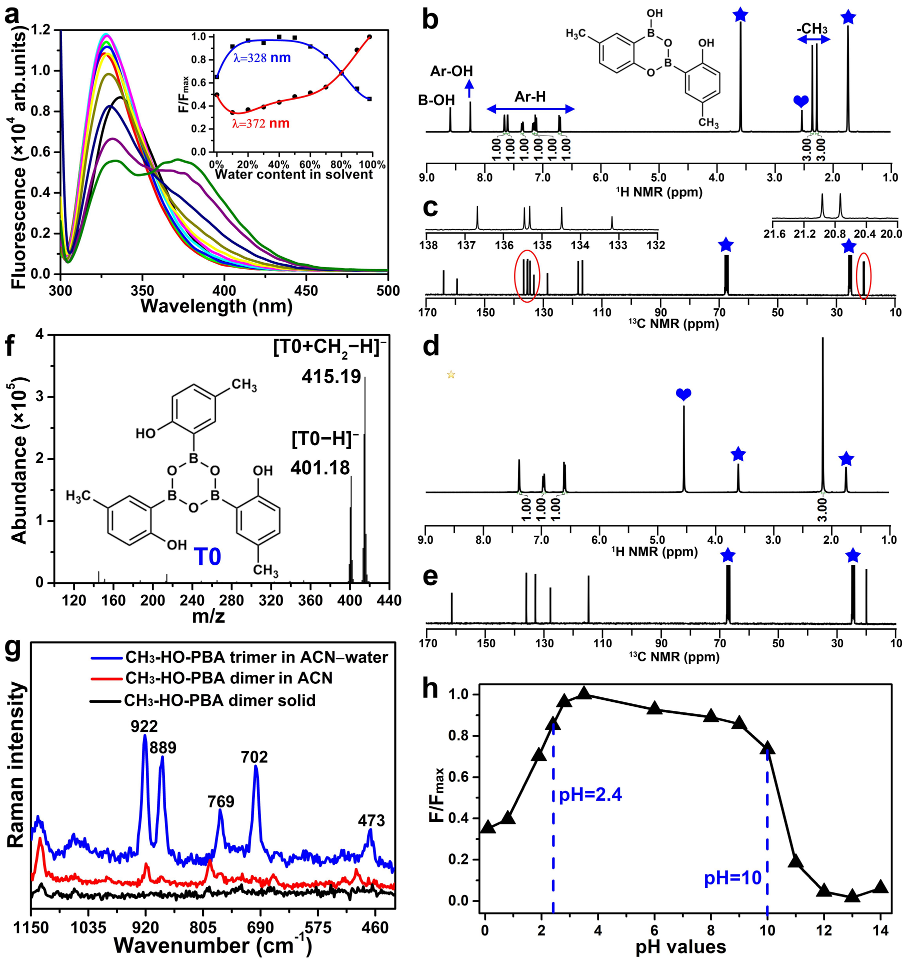

Supplementary Fig. 13 Transformation of CH_3-HO-PBA dimer into trimer upon exposure to water. a Fluorescence spectra and intensity changes (at 328 and 372 nm, inset) of CH_3-HO-PBA dimer (2×10^{-5} mol·L^{-1}) in THF–water mixtures with different amounts of water (volume%), λ_{ex}=298 nm. **b-e** ^{1}H (**b**, **d**) and ^{13}C (**c**, **e**) NMR spectra of CH_3-HO-PBA dimer in THF−d_8 (**b**, **c**) and in D_2O–THF−d_8 (2:1, v/v) mixture (**d**, **e**) at room temperature, concentration: 8 mg·mL^{-1}.The signals with heart and star mark correspond to H_2O and THF, respectively. **f** ESI-Q-TOF mass spectrum of CH_3-HO-PBA dimer in the methanol–water (5:1, v/v) solution, acquired in a negative mode. **g** UV–Raman spectra of the solid CH_3-HO-PBA dimer sample (black), CH_3-HO-PBA dimer in a dry ACN solution (red) and CH_3-HO-PBA trimer in an ACN–water (1:2, v/v) solution (blue). **h** Relative fluorescence intensity at 372 nm of CH_3-HO-PBA trimer (F/F_{max}) as a function of pH value.

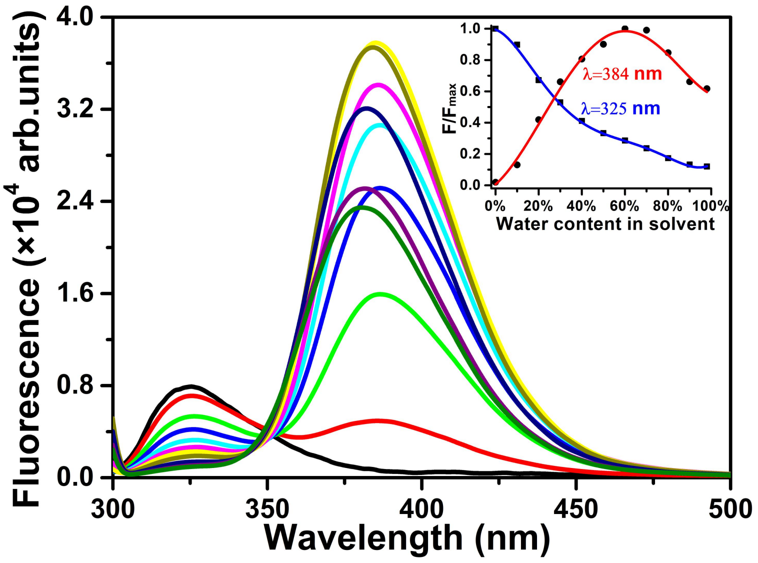

Supplementary Fig. 14 Fluorescence spectra and intensity changes (at 325 and 384 nm, inset) of CF_3-HO-PBA dimer (2×10^{-5} mol·L^{-1}) in THF–water mixtures with different amounts of water (volume%), λ_{ex}=295 nm.

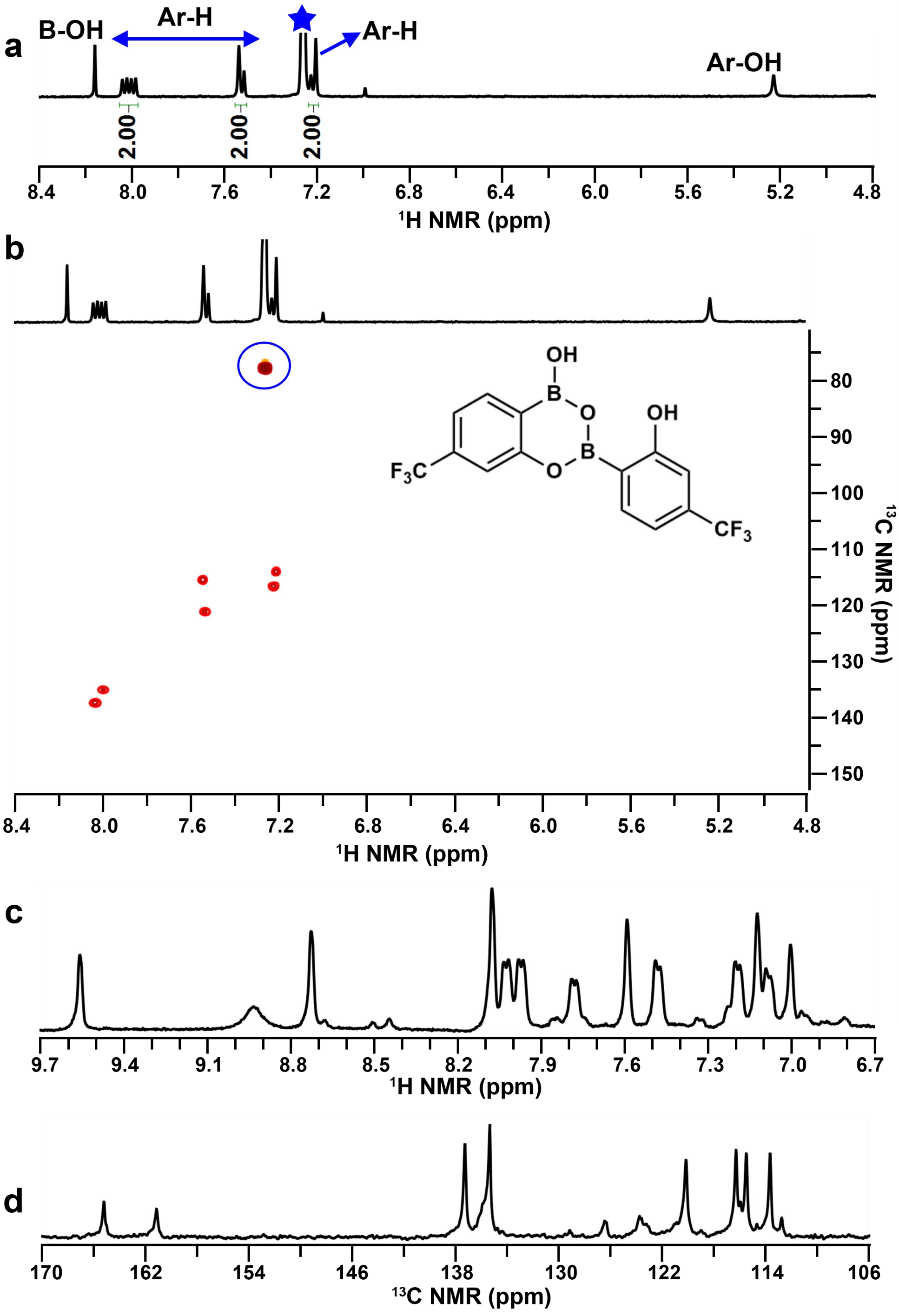

Supplementary Fig. 15 NMR spectra of CF_3-HO-PBA dimer in $CDCl_3$ and THF−d_8. a,b ^{1}H (**a**) and ^{1}H -^{13}C HSQC (**b**) NMR spectra of CF_3-HO-PBA dimer in $CDCl_3$ at room temperature. The signal with star mark (**a**) and that enclosed in blue circle (**b**) correspond to $CHCl_3$. **c,d** ^{1}H (**c**) and ^{13}C (**d**) NMR spectra of CF_3-HO-PBA dimer in THF−d_8 at room temperature, concentrations: 8 mg·mL^{-1}.

Discussion: In $CDCl_3$, CF_3-HO-PBA dimer exhibits six sets of signals in its ^1H NMR spectrum (Supplementary Fig. 15a). The low solubility of CF_3-HO-PBA dimer in $CDCl_3$ makes it challenging to obtain a clear ^{13}C NMR spectrum. As an alternative, a ^1H-^{13}C HSQC spectrum with higher sensitivity was measured, which displays six cross peaks (Supplementary Fig. 15b). The numbers of signals in both ^1H NMR and ^1H-^{13}C HSQC spectrum of CF_3-HO-PBA dimer in $CDCl_3$ are consistent with the numbers of H atoms present in CF_3-HO-PBA dimer. However, when CF_3-HO-PBA dimer was dissolved in THF−d_8, both ^1H and ^{13}C NMR spectrum display more sets of signals than the numbers of H and C atoms in CF_3-HO-PBA dimer (Supplementary Fig. 15c,d). That is because a portion of CF_3-HO-PBA dimer transforms into CF_3-HO-PBA trimer due to the presence of a small amount of water in THF−d_8 (Supplementary Fig. 13b), resulting in the coexisting of CF_3-HO-PBA dimer and trimer. Upon addition of water into this THF−d_8 solution, CF_3-HO-PBA dimer completely transforms into CF_3-HO-PBA trimer. Consequently, the numbers of ^1H and ^{13}C NMR signals in CF_3-HO-PBA dimer are reduced to three and five sets, respectively (Supplementary Fig. 16). Unlike CF_3-HO-PBA dimer, both HO-PBA dimer (Fig. 3b,c in text) and CH_3-HO-PBA dimer (Supplementary Fig. 13b,c) remain stable in THF−d_8, despite the presence of a small amount of water in THF−d_8 (Supplementary Fig. 13b). This result indicates that electron-withdrawing group of −CF_3 improves the sensitivity of the dimeric structure to water.

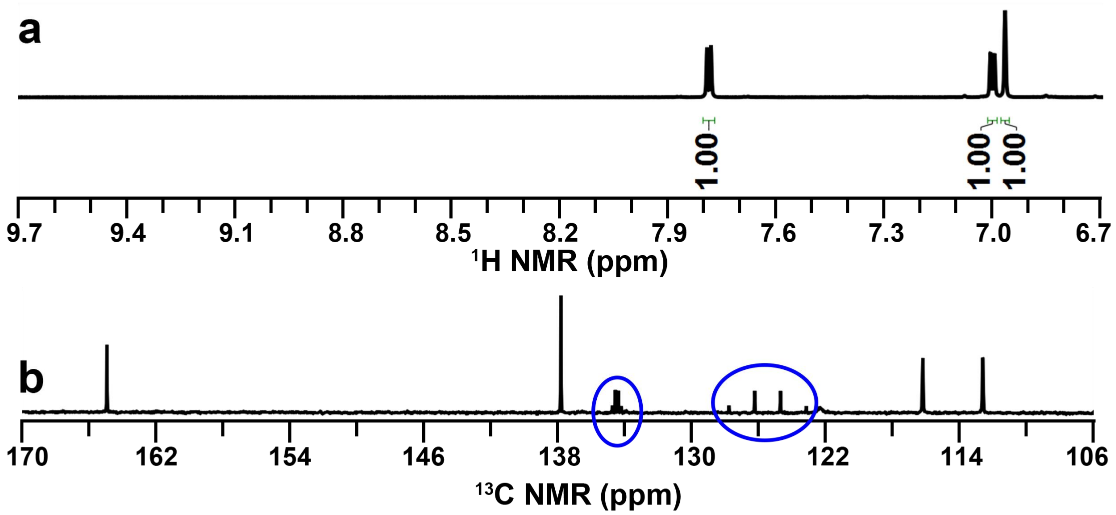

Supplementary Fig. 16 a,**b** ^{1}H (**a**) and ^{13}C (**b**) NMR spectra of CF_3-HO-PBA dimer in D_2O–THF−d_8 (2:1, v/v) mixture at room temperature, concentrations: 8 mg·mL^{-1}.

Discussion: The two quartets enclosed in blue circle arise (Supplementary Fig. 16b) from the C-F coupling. The first quartet around 125.5 ppm with a large J-coupling corresponds to the carbon of the –CF_3 group, and the second quartet around 134.5 ppm corresponds to the carbon bonded to the –CF_3 group.

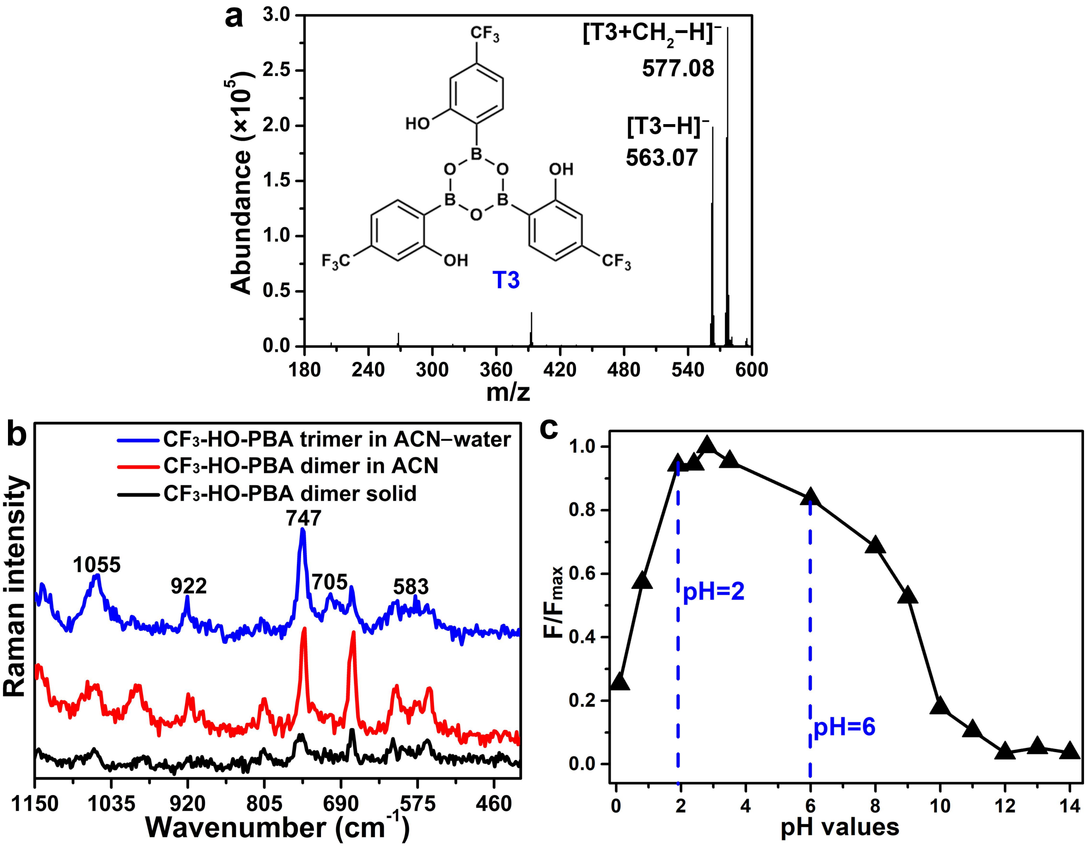

Supplementary Fig. 17 CF_3-HO-PBA trimer and its structure characterization. a ESI-Q-TOF mass spectrum of CF_3-HO-PBA dimer in the methanol–water (5:1, v/v) solution, acquired in a negative mode. **b** UV–Raman spectra of the solid CF_3-HO-PBA dimer sample (black), CF_3-HO-PBA dimer in a dry ACN solution (red) and CF_3-HO-PBA trimer in an ACN–water (1:2, v/v) solution (blue). **c** Relative fluorescence intensity at 384 nm of CF_3-HO-PBA trimer (F/F_{max}) as a function of pH value.

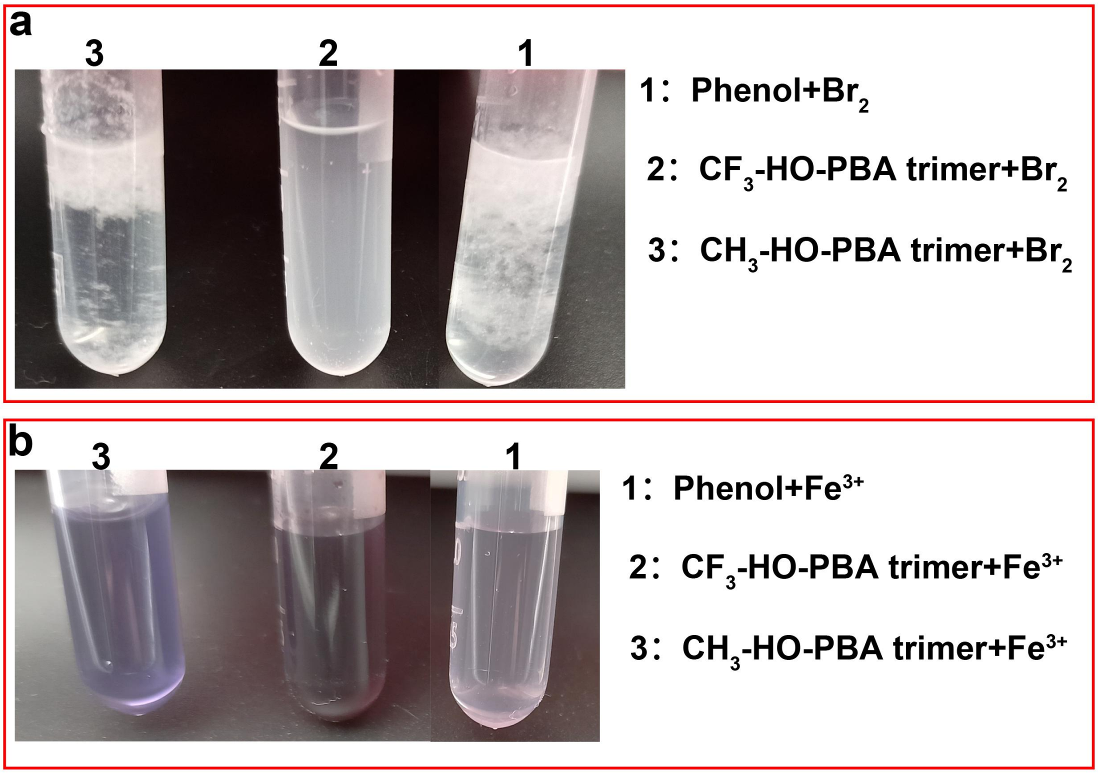

Supplementary Fig. 18 a,**b** Photos of the phenol, CF_3-HO-PBA trimer and CH_3-HO-PBA trimer solution after addition of Br_2 (**a**) and $FeCl_3$ (**b**).

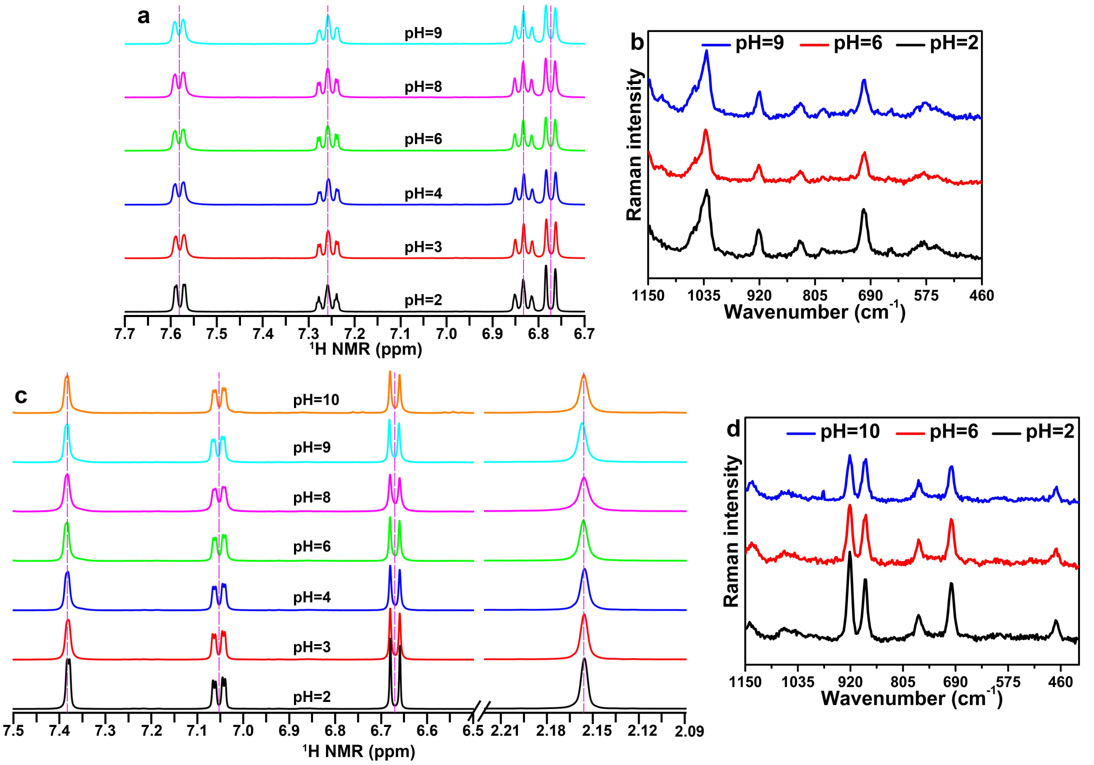

Supplementary Fig. 19 Stability of the boroxine structure at various pH values. a, c ^{1}H NMR spectra of HO-PBA trimer (a) and CH_3-HO-PBA trimer (c) at different pH values in a D_2O–ethanol–d_6 (1:1, v/v) solution. **b, d** UV–Raman spectra of HO-PBA trimer (b) and CH_3-HO-PBA trimer (d) at different pH values in an ACN–water (1:2, v/v) solution.

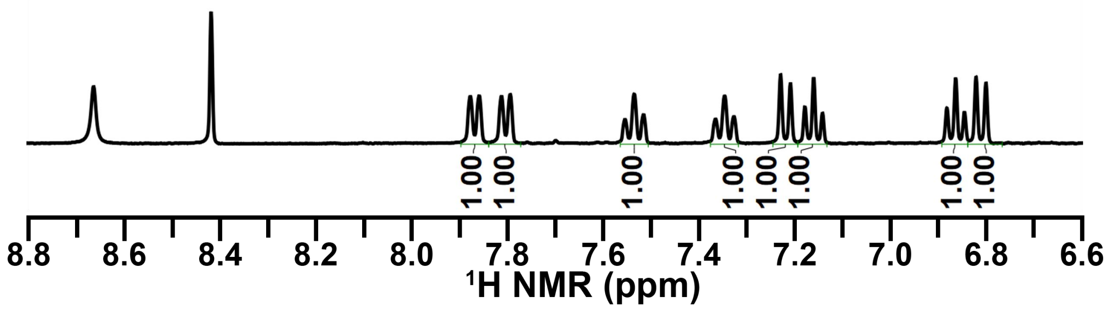

Supplementary Fig. 20 ^{1}H NMR spectrum of product in THF−d_8 from lyophilization of the THF–water (1:2, v/v) solution of HO-PBA trimer at room temperature, concentrations: 8 mg·mL^{-1}.

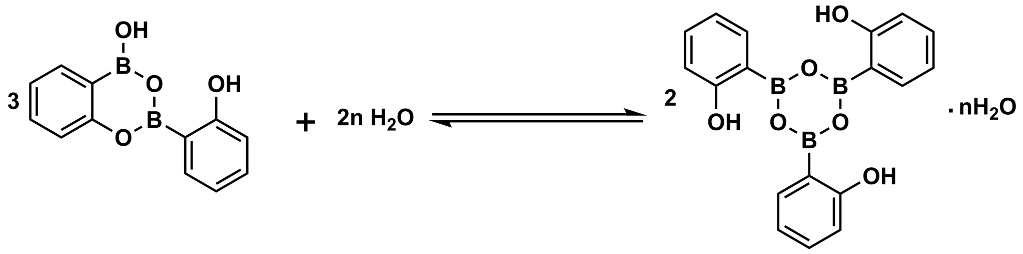

Supplementary Fig. 21 Reversible reaction between HO-PBA dimer and H_2O to produce HO-PBA trimer–H_2O complex.

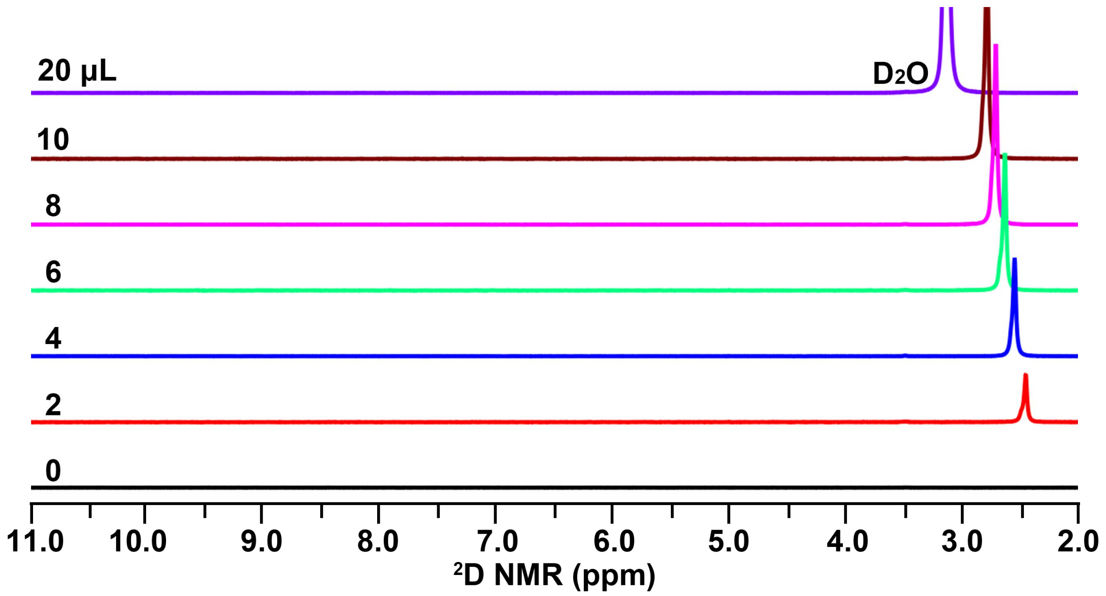

Supplementary Fig. 22 ^{2}D NMR spectra of THF with various amount of D_2O.

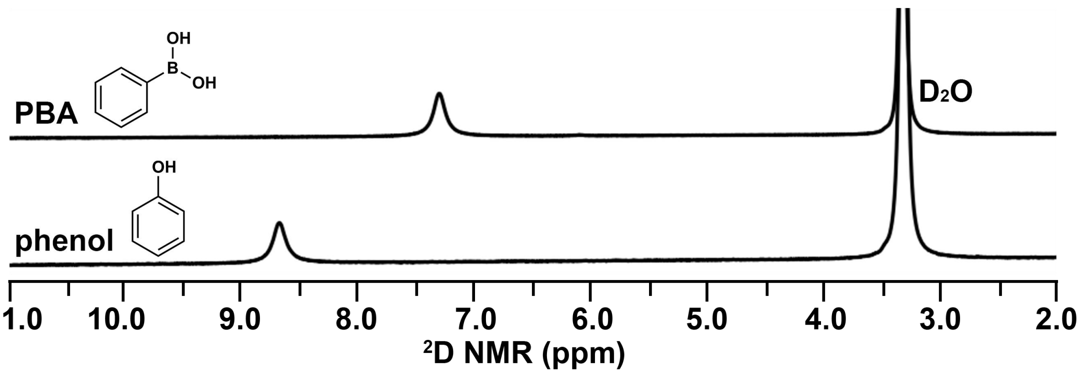

Supplementary Fig. 23 ^{2}D NMR spectra of PBA (0.27 mol·L^{-1}) and phenol (0.27 mol·L^{-1}) in a THF–D_2O (25:1, v/v) solution.

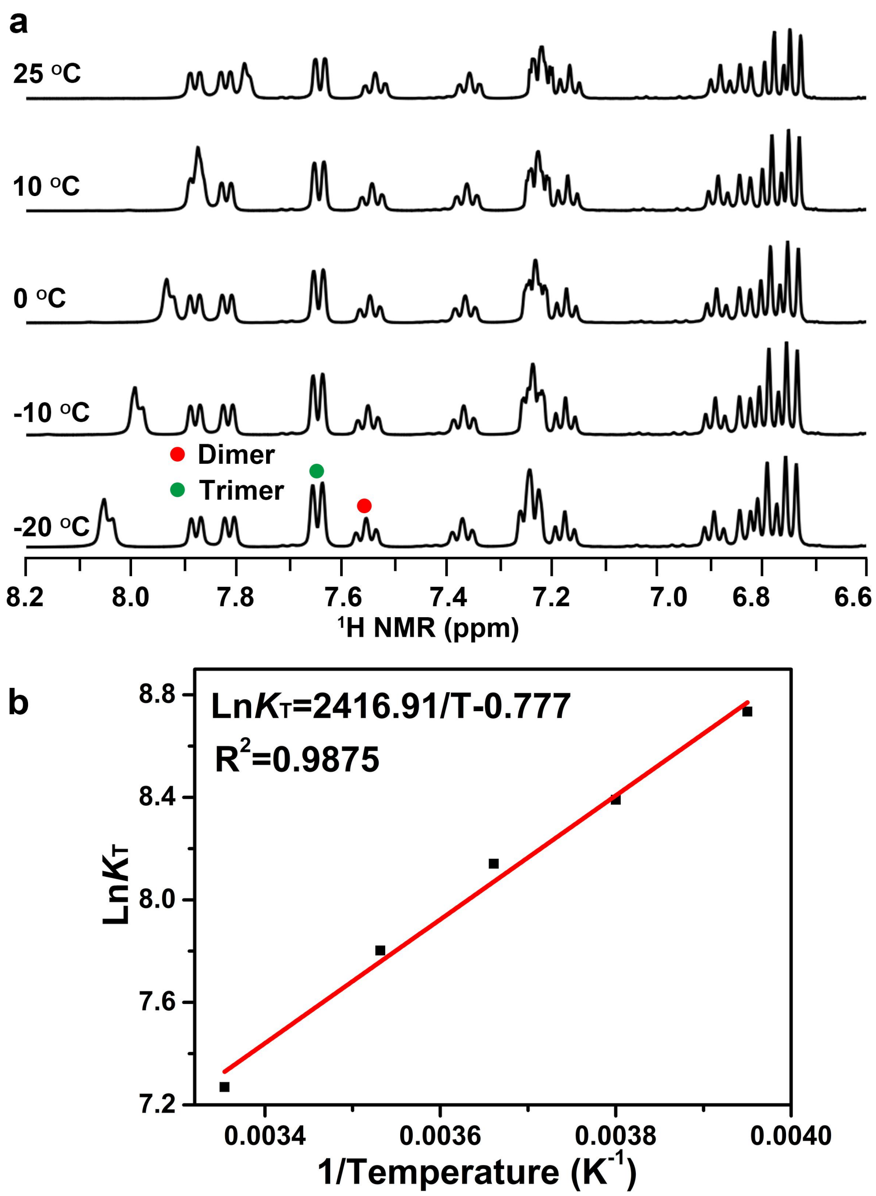

Supplementary Fig. 24 Determination of thermodynamic parameters for the transformation from HO-PBA dimer to HO-PBA trimer–D_2O complex. a VT-^{1}H NMR spectra of equilibrium mixture of HO-PBA dimer and trimer, which was prepared by dissolving 32.8 mg HO-PBA dimer in a D_2O (4.5 μL) and THF−d_8 (500 μL) mixture. **b** van't Hoff plot of the transformation from HO-PBA dimer to trimer.

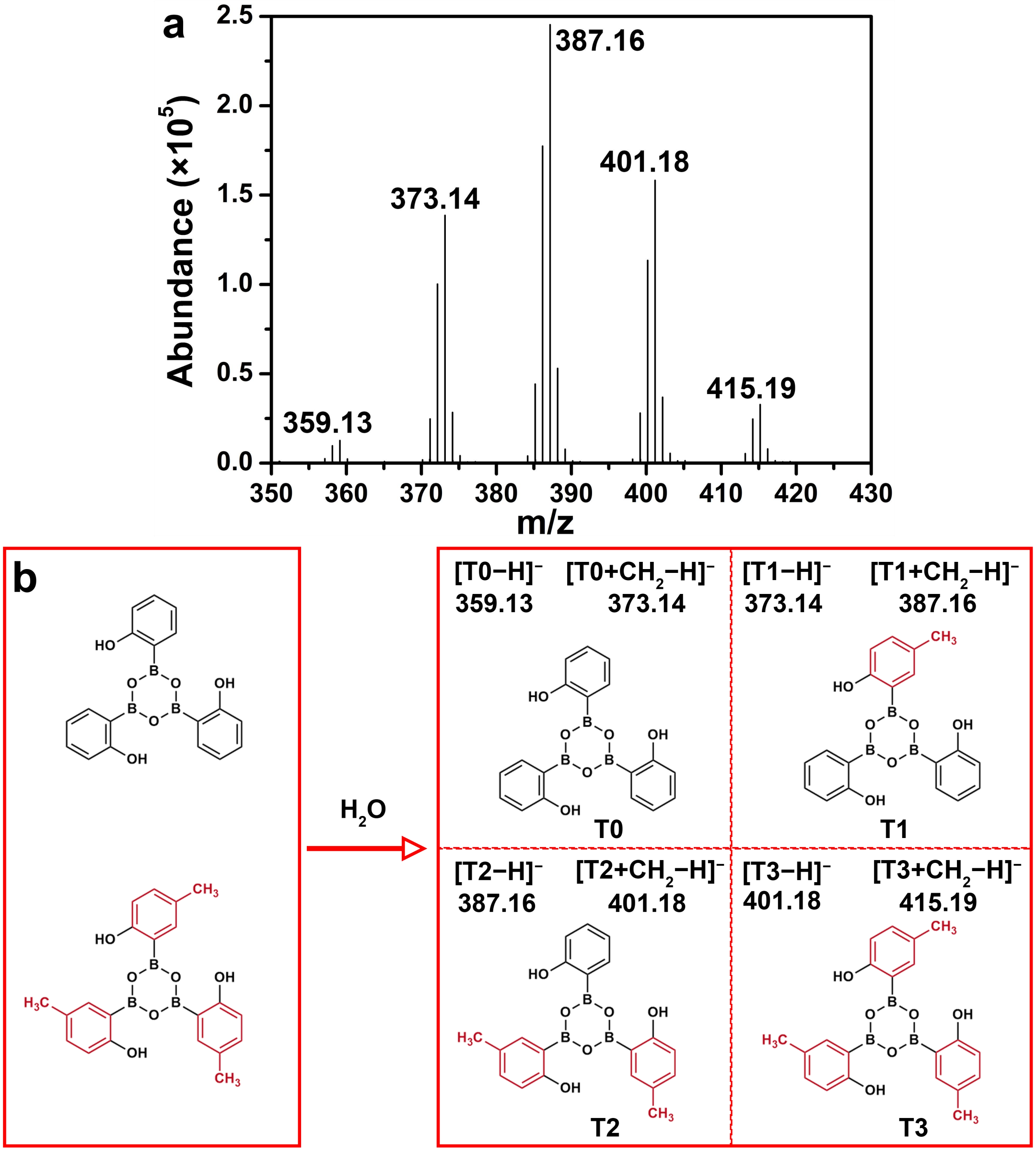

Supplementary Fig. 25 Exchange reaction between HO-PBA trimers and CH_3-HO-PBA trimers. a ESI-Q-TOF mass spectrum of products from mixing HO-PBA dimers and CH_3-HO-PBA dimers together in the methanol–water (5:1, v/v) solution, acquired in a negative mode. **b** Illustration of the dynamic exchange between HO-PBA trimer and CH_3-HO-PBA trimer.

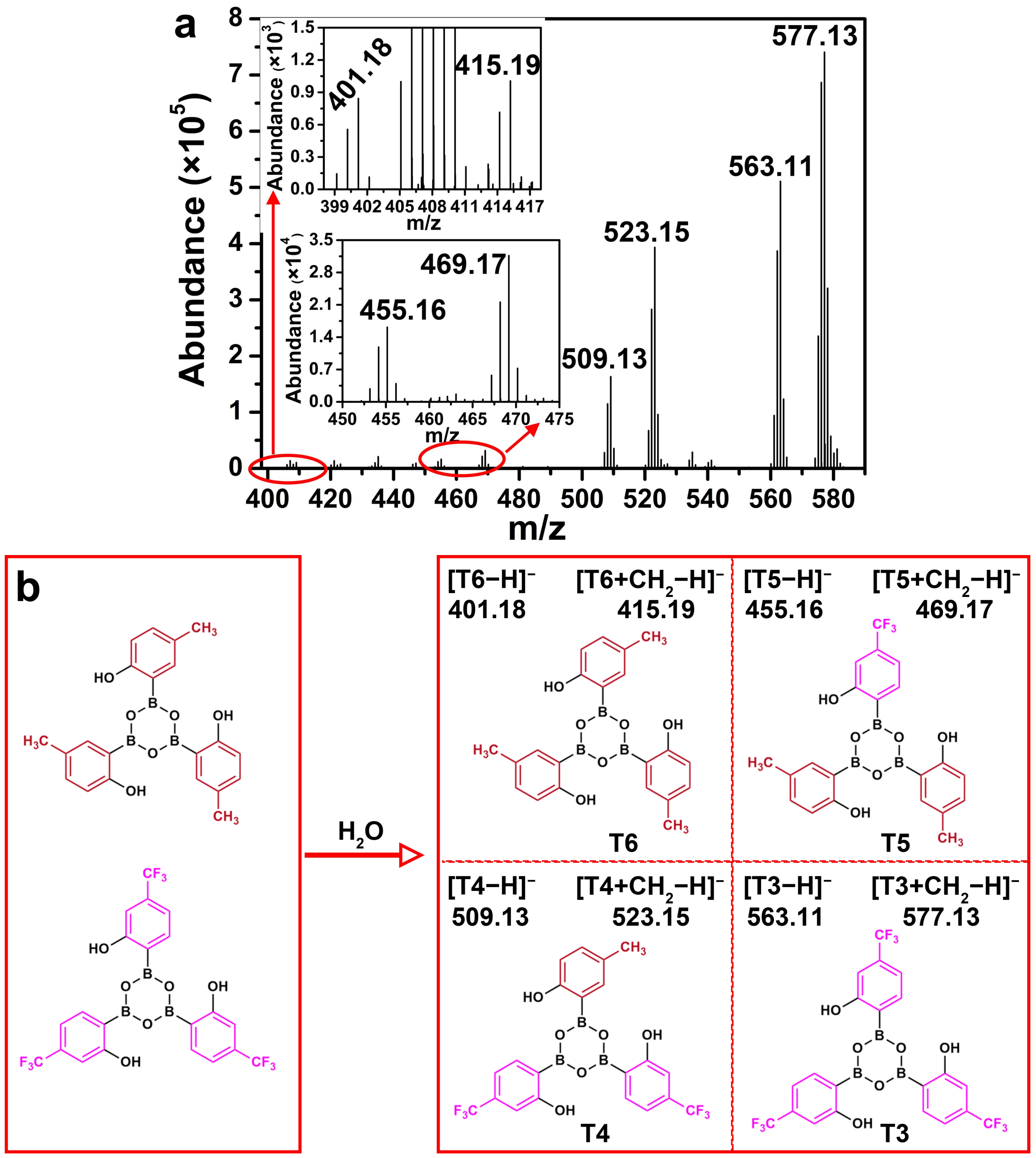

Supplementary Fig. 26 Exchange reaction between CF_3-HO-PBA trimers and CH_3-HO-PBA trimers. a ESI-Q-TOF mass spectrum of products from mixing CF_3-HO-PBA dimers and CH_3-HO-PBA dimers together in the methanol–water (5:1, v/v) solution, acquired in a negative mode. **b** Illustration of the dynamic exchange between CF_3-HO-PBA trimer and CH_3-HO-PBA trimer.

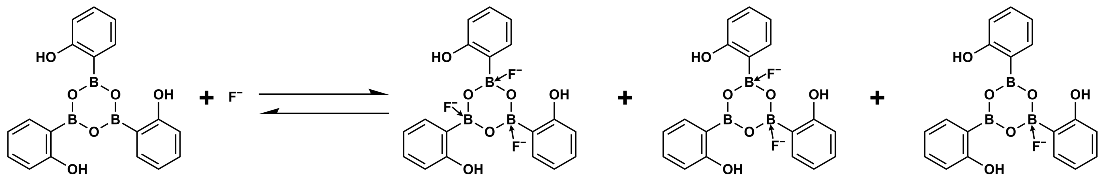

Supplementary Fig. 27 Equilibration between HO-PBA trimer–F⁻ complexes and HO-PBA trimer.

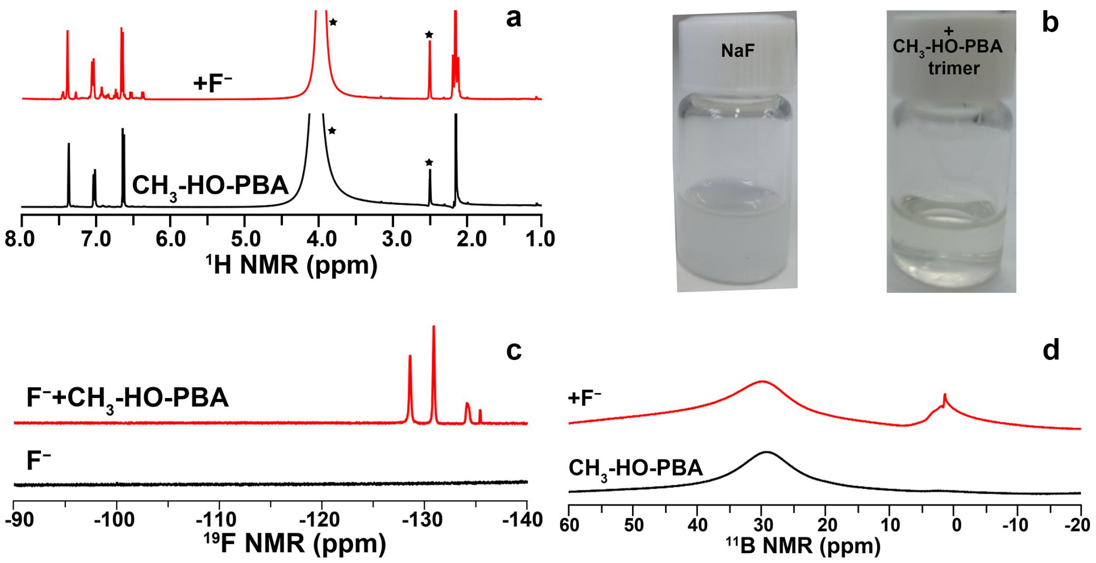

Supplementary Fig. 28 Ultra-strong binding capacity of CH_3-HO-PBA trimer toward F^- in aqueous media. a ^{1}H NMR spectra of CH_3-HO-PBA trimer (120 mmol·L^{-1}) before and after the addition of one molar equivalent of NaF. The signals with star mark correspond to DMSO (2.5 ppm) and H_2O, respectively. **b**,**c** Photos (**b**) and ^{19}F NMR spectra (**c**) of NaF (120 mmol·L^{-1}) before and after addition of one molar equivalent of CH_3-HO-PBA trimer. **d** ^{11}B NMR spectra of CH_3-HO-PBA trimer (120 mmol·L^{-1}) before and after addition of one molar equivalent NaF. All NMR measurements were carried out at room temperature using D_2O–DMSO–d_6 (1:6, v/v) mixtures as solvents.

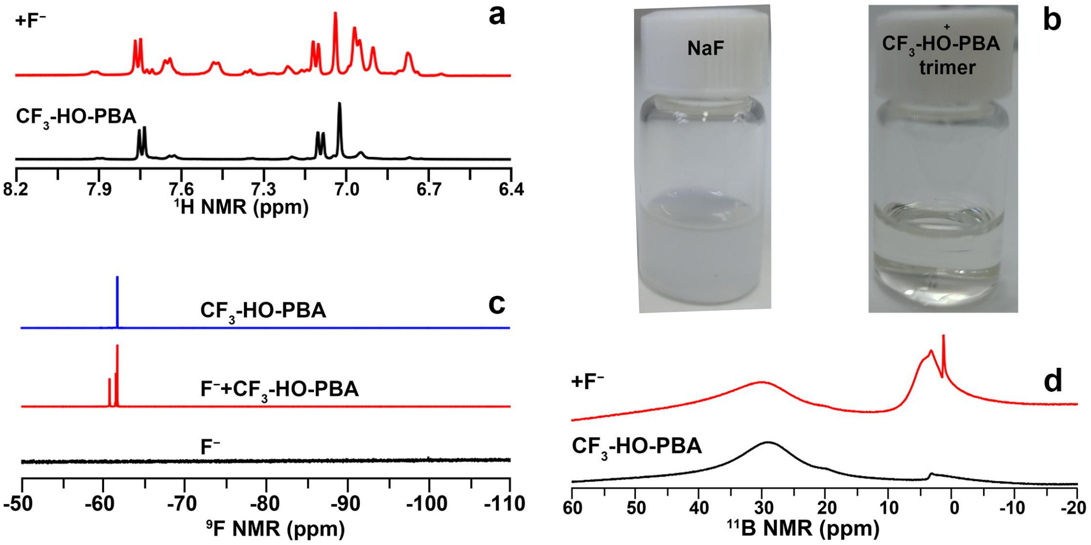

Supplementary Fig. 29 Ultra-strong binding capacity of CF_3-HO-PBA trimer toward F^- in aqueous media. a ^1H NMR spectra of CF_3-HO-PBA trimer (120 mmol·L^{-1}) before and after the addition of one molar equivalent of NaF. **b**,**c** Photos (**b**) and ^{19}F NMR spectra (**c**) of NaF (120 mmol·L^{-1}) before and after addition of one molar equivalent of CF_3-HO-PBA trimer. **d** ^{11}B NMR spectra of CF_3-HO-PBA trimer (120 mmol·L^{-1}) before and after addition of one molar equivalent NaF. All NMR measurements were carried out at room temperature using D_2O–DMSO–d_6 (1:6, v/v) mixtures as solvents.

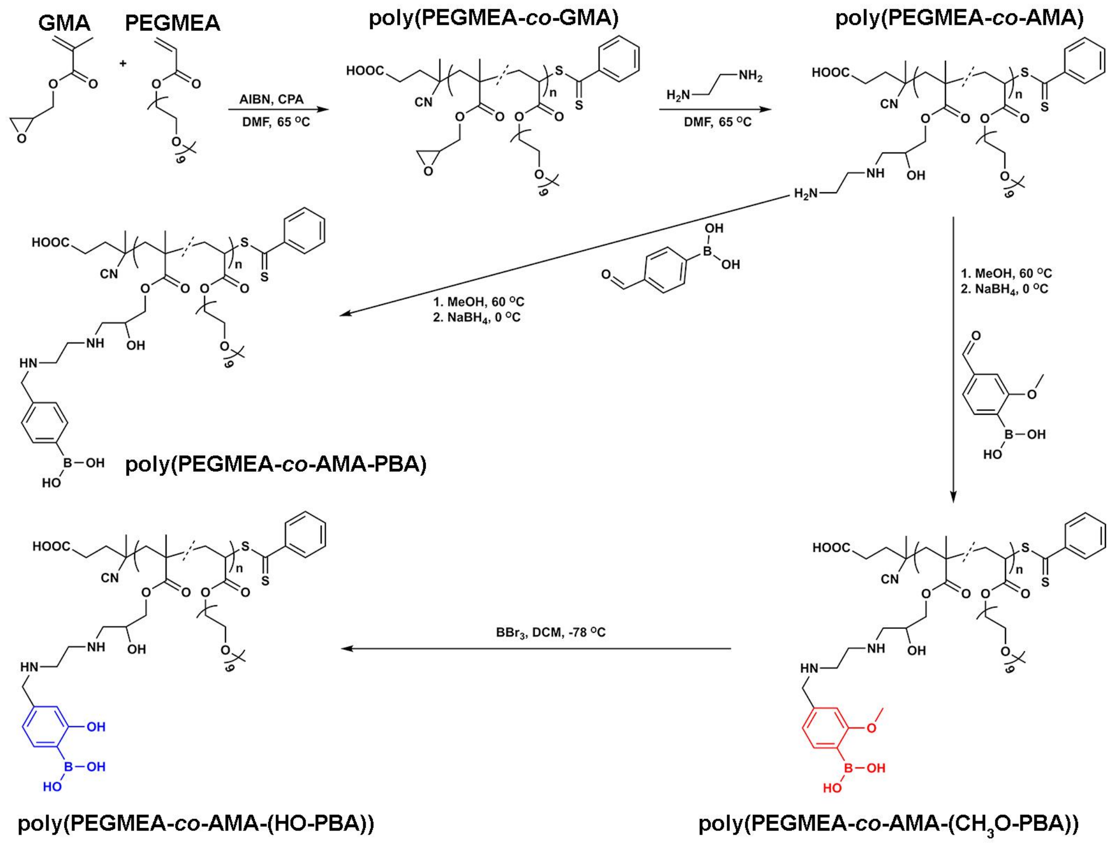

Supplementary Fig. 30 Synthesis of poly(PEGMEA-*co*-GMA), poly(PEGMEA-*co*-AMA), poly(PEGMEA-*co*-AMA-PBA), poly(PEGMEA-*co*-AMA-(CH_3O-PBA)) and poly(PEGMEA-*co*-AMA-(HO-PBA)).

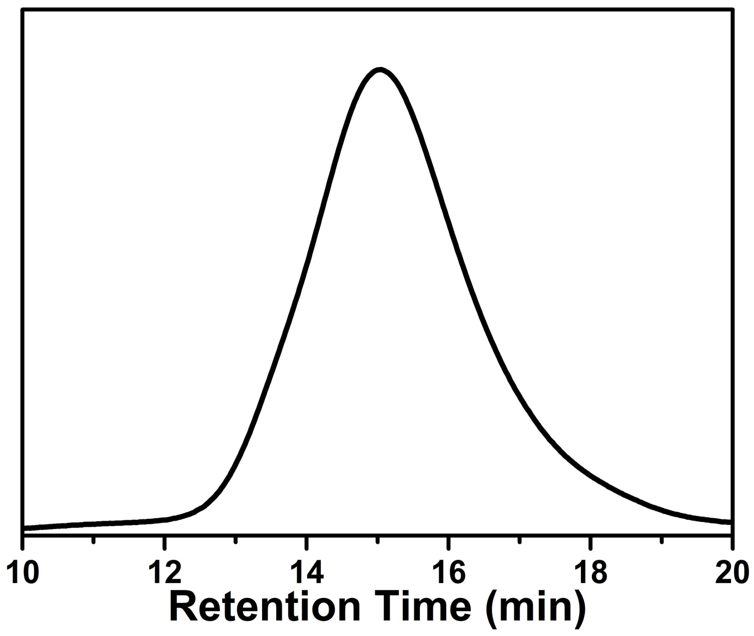

Supplementary Fig. 31 GPC profiles of poly(PEGMEA-*co*-GMA).

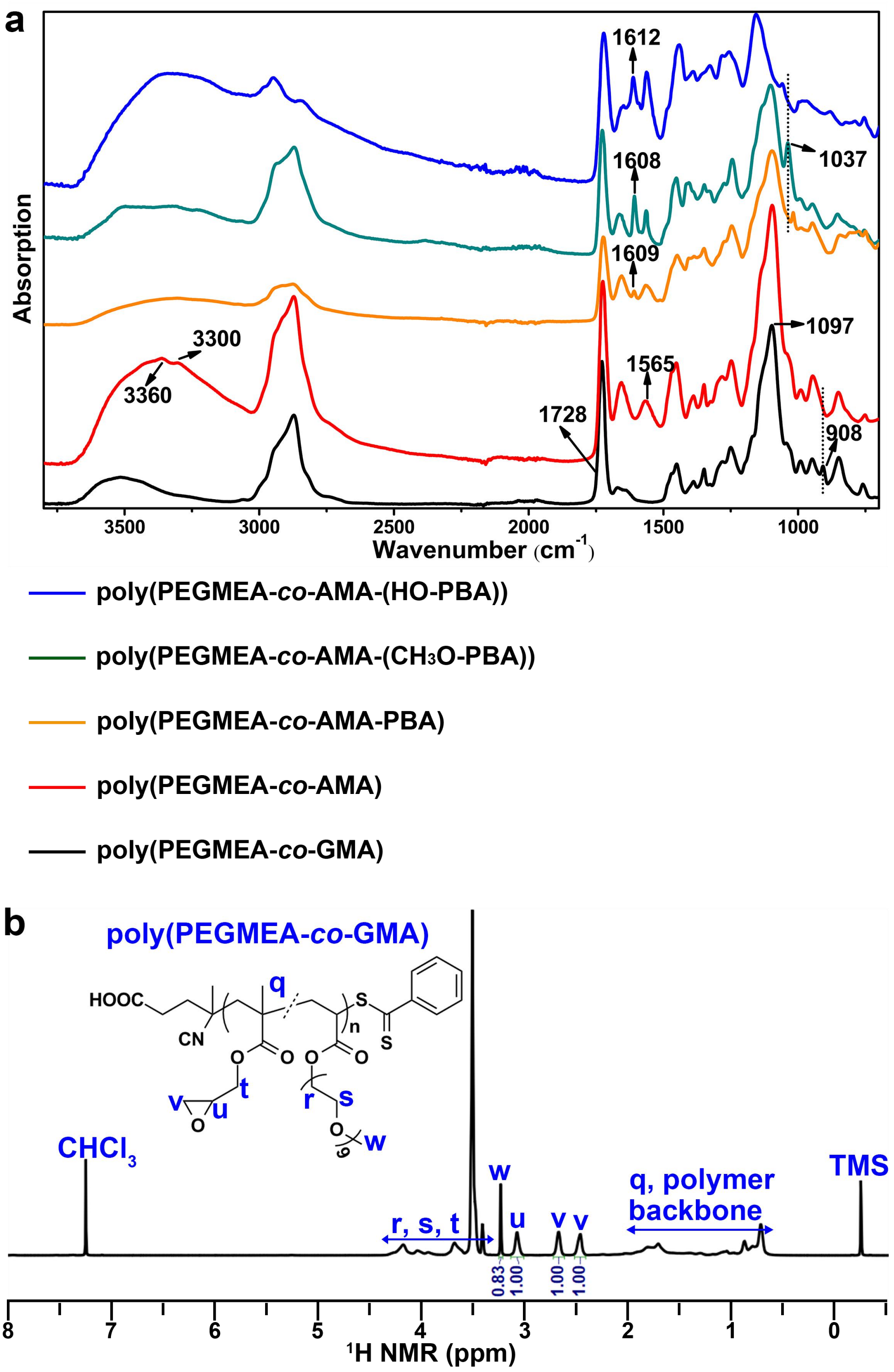

Supplementary Fig. 32 Characterization of polymers. **a** ATR-FTIR spectra of poly(PEGMEA-*co*-GMA) (black), poly(PEGMEA-*co*-AMA) (red), poly(PEGMEA-*co*-AMA-PBA) (orange), poly(PEGMEA-*co*-AMA-(CH_3O-PBA)) (cyan) and poly(PEGMEA-*co*-AMA-(HO-PBA)) (blue). **b** ^{1}H NMR spectra of poly(PEGMEA-*co*-GMA) in $CDCl_3$ at room temperature.

Discussion: In the ATR-FTIR spectrum of poly(PEGMEA-*co*-GMA) (Supplementary Fig. 32a, black line), the peak at 908 cm^{-1} corresponds to the epoxy groups of GMA, the peak at 1728 cm^{-1} is ascribed to the stretching vibrations of the ester groups in both GMA and PEGMEA[1-3], and the peak at 1097 cm^{-1} originates from the stretching vibration of the ether bonds of PEGMEA. These peaks collectively indicate the presence of both GMA and PEGMEA fragments within the copolymer, indicating the successful synthesis of poly(PEGMEA-*co*-GMA). In the ^1H NMR spectra of poly(PEGMEA-*co*-GMA) (Supplementary Fig. 32b), the signals at 2.62 (v) and 2.82 (v) ppm attribute to –CH_2–of the epoxy ring in GMA unit. The signals at 3.21 (u) and 3.37 (w) ppm attribute to –CH– of the epoxy ring in GMA unit and –CH_3 in PEGMEA unit, respectively[1,4]. By calculating the ratio of the integral of the signal at 3.21 (u) to that at 3.37 (w) ppm, the molar ratio of GMA to PEGMEA was determined to be 1:0.28 (poly($PEGMEA_{0.28m}$-*co*-GMA_m)).

Upon reacting with ethylenediamine, the peak at 908 cm^{-1} associated with epoxy vibration disappears, and three new bands originating from N-H vibrations appear at 3360, 3300 and 1565 cm^{-1} in the ATR-FTIR spectrum of poly(PEGMEA-*co*-AMA) (Supplementary Fig. 32a, red line). These changes suggest the complete reaction of the epoxy group with ethylenediamine in the synthesis of poly(PEGMEA-*co*-AMA). Thus, the molar ratio of AMA to PEGMEA in poly(PEGMEA-*co*-AMA) is also 1:0.28 (poly($PEGMEA_{0.28m}$-*co*-AMA_m)).

In the ATR-FTIR spectrum of poly(PEGMEA-*co*-AMA-PBA) (Supplementary Fig. 32a, orange line), a peak corresponding to the aromatic C=C stretching vibration is observed at 1609 cm^{-1}, indicating the successful functionalization of PBA into poly(PEGMEA-*co*-AMA). The mass percentage of boron in poly(PEGMEA-*co*-AMA-PBA) was determined to be 1.49% using ICP–OES. Based on the boron content, the molar ratio of AMA to PBA in poly(PEGMEA-*co*-AMA-PBA) was calculated to be ca. 1:0.57 (poly($PEGMEA_{0.28m}$-*co*-AMA_m-$PBA_{0.57m}$).

In the ATR-FTIR spectrum of poly(PEGMEA-*co*-AMA-(CH_3O-PBA)) (Supplementary Fig. 32a, cyan line), the appearance of peaks at 1608 cm^{-1}, corresponding to the aromatic C=C stretching vibration, and at 1037 cm^{-1}, corresponding to the stretching vibration of the Ar-O-C groups, provides strong evidences for the successful functionalization of CH_3O-PBA into poly(PEGMEA-*co*-AMA). After reacting poly(PEGMEA-*co*-AMA-(CH_3O-PBA)) with BBr_3, the aromatic C=C stretching vibration shifts to 1612 cm^{-1}, as shown in the ATR-FTIR spectrum of

poly(PEGMEA-*co*-AMA-(HO-PBA) (Supplementary Fig. 32a, blue line). Moreover, the stretching vibration of the Ar-O-C groups at 1037 cm^{-1} disappears, indicating the complete conversion of poly(PEGMEA-*co*-AMA-(CH_3O-PBA)) into poly(PEGMEA-*co*-AMA-(HO-PBA).

The mass percentages of boron in poly(PEGMEA-*co*-AMA-(HO-PBA)) was determined to be 1.06% using ICP–OES. Based on the boron content, the molar ratio of AMA to HO-PBA in poly(PEGMEA-*co*-AMA-(HO-PBA)) was calculated to be ca. 1:0.39 (poly($PEGMEA_{0.28m}$-*co*-AMA_m-$(HO\text{-}PBA)_{0.39m}$). The molar ratio of CH_3O-PBA to AMA in poly(PEGMEA-*co*-AMA-(CH_3O-PBA)) is also 1:0.39 (poly($PEGMEA_{0.28m}$-*co*-AMA_m-$(CH_3O\text{-}PBA)_{0.39m}$)), since (poly($PEGMEA_{0.28m}$-*co*-$AMA_m$-$(HO\text{-}PBA)_{0.39m}$ was synthesized by adopting BBr_3 to convert methoxy groups of CH_3O-PBAs in poly(PEGMEA-*co*-AMA-(CH_3O-PBA)) into hydroxyl groups.

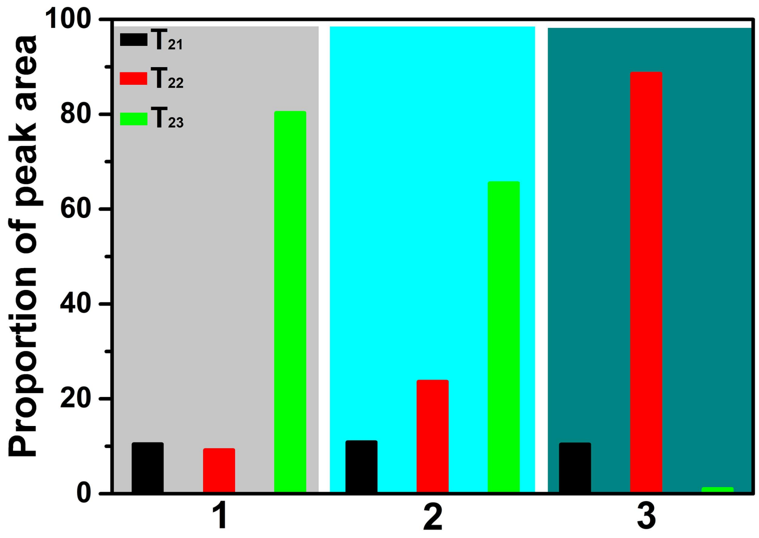

Supplementary Fig. 33 The proportion of peak areas of the three peaks, T_{21}, T_{22} and T_{23}, in T_2 distribution curves of poly(PEGMEA-*co*-AMA-PBA) (**1**), poly(PEGMEA-*co*-AMA-(CH_3O-PBA)) (**2**) and poly(PEGMEA-*co*-AMA-(HO-PBA)) (**3**) in water at room temperature.

Supplementary Tables 1–11

Supplementary Table 1 Energy change for the dehydration of HO-PBA monomer to HO-PBA dimer, calculated by density functional theory with M06-2X functional and TZVP basis sets

Substances	HO-PBA monomer (A) 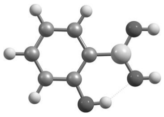	HO-PBA dimer (B) 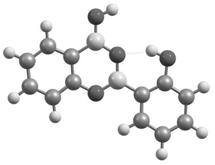	H_2O 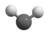	$2A \rightarrow B+2H_2O$
Gibbs Free Energy (kJ·mol^{-1})	–1269134.9	–2137012.4	–200639.4	–21.4

Supplementary Table 2 Crystal data and structure refinements for HO-PBA

Empirical formula	$C_{12}H_{10}B_2O_4$
Formula weight	239.82
Temperature (K)	120
Crystal size (mm^3)	0.16 x 0.12 x 0.12
Crystal system	monoclinic
Space group	C 12/c 1
a (Å)	25.308(5)
b (Å)	5.0812(10)
c (Å)	17.183(4)
α (°)	90
β (°)	91.498(9)
γ (°)	90
V (Å^3)	2208.9(8)
Z	8
Density (g/cm^3)	1.442
F (000)	992.0
Theta range for data collection	2.83 to 27.18
Index ranges	−32<=h<=32, −6<=k<=6, −22<=l<=22
Reflections collected	18588
Independent reflections	2557 [R(int)= 0.0681]
Final R indices [I>2sigma(I)]	R1= 0.0476, wR2=0.1056
R indices (all data)	R1= 0.0764, wR2= 0.1198
Goodness−of−fit on F^2	1.034
Highest difference peak and deepest hole (e Å^{-3})	0.254 and -0.224

Supplementary Table 3 Bond lengths (Å) and angles for HO-PBA

O2−B2	1.379(2)	C11−C12−O4	116.04(15)
O2−B1	1.407(2)	C11−C12−C7	122.32(16)
O3−C1	1.389(2)	C1−C2−C3	117.37(16)
O3−B2	1.370(2)	C1−C2−B1	117.76(15)
O1−H1	0.8400	C3−C2−B1	124.87(16)
O1−B1	1.338(2)	C12−C7−B2	122.88(15)
O4−H4	0.8400	C8−C7−C12	116.39(16)
O4−C12	1.387(2)	C8−C7−B2	120.72(16)
C1−C2	1.394(2)	C12−C11−H11	120.3
C1−C6	1.388(2)	C10−C11−C12	119.43(17)
C12−C7	1.402(2)	C10−C11−H11	120.3
C12−C11	1.382(2)	C2−C3−H3	119.5
C2−C3	1.406(2)	C4−C3−C2	120.94(16)
C2−B1	1.545(3)	C4−C3−H3	119.5
C7−C8	1.402(2)	C7−C8−H8	119
C7−B2	1.552(3)	C9−C8−C7	122.02(17)
C11−H11	0.9500	C9−C8−H8	119
C11−C10	1.379(3)	C1−C6−H6	120.8
C3−H3	0.9500	C5−C6−C1	118.38(16)
C3−C4	1.387(2)	C5−C6−H6	120.8
C8−H8	0.9500	C3−C4−H4A	120.2
C8−C9	1.388(3)	C3−C4−C5	119.55(16)
C6−H6	0.9500	C5−C4−H4A	120.2
C6−C5	1.381(2)	C6−C5−C4	121.22(16)
C4−H4A	0.9500	C6−C5−H5	119.4
C4−C5	1.388(2)	C4−C5−H5	119.4
C5−H5	0.9500	C11−C10−H10	119.7
C10−H10	0.95	C11−C10−C9	120.53(17)

C10−C9	1.385(3)	C9−C10−H10	119.7
C9−H9	0.95	C8−C9−H9	120.4
B2−O2−B1	121.16(14)	C10−C9−C8	119.29(17)
B2−O3−C1	121.78(14)	C10−C9−H9	120.4
B1−O1−H1	109.5	O2−B2−C7	119.49(15)
C12−O4−H4	109.5	O3−B2−O2	121.77(16)
O3−C1−C2	120.73(15)	O3−B2−C7	118.74(16)
C6−C1−O3	116.73(15)	O2−B1−C2	116.73(15)
C6−C1−C2	122.54(16)	O1−B1−O2	120.43(16)
O4−C12−C7	121.63(15)	O1−B1−C2	122.83(16)

Supplementary Table 4 Hydrogen bond data for HO-PBA

D−H...A	d(D−H)	d(H...A)	d(D...A)	<(DHA)
O4−H4...O2	0.8401 (14)	1.9355 (11)	2.6926 (19)	149.371 (105)
O1-H1...O4^{i}	0.8401 (14)	1.9755 (15)	2.7982 (21)	166.092 (105)
C9−H9...O3^{ii}	0.9500 (18)	3.0058 (12)	3.6799 (22)	129.128 (110)

Symmetry codes: (i) 0.5−*x*, 0.5+*y*, 0.5−*z*; (ii) *x*, 2-*y*, -0.5+*z*.

Supplementary Table 5 Crystal data and structure refinements for CH_3-HO-PBA

Empirical formula	$C_{14}H_{14}B_2O_4$
Formula weight	267.87
Temperature (K)	120
Crystal size (mm^3)	0.15 x 0.12 x 0.12
Crystal system	monoclinic
Space group	P 1 21/n 1
a (Å)	12.8607(14)
b (Å)	5.0210(5)
c (Å)	19.993(2)
α (°)	90
β (°)	91.001(5)
γ (°)	90
V (Å^3)	1290.8(2)
Z	4
Density (g/cm^3)	1.378
F (000)	560.0
Theta range for data collection	3.17 to 27.17
Index ranges	−16<=h<=16, −6<=k<=6, −25<=l<=25
Reflections collected	15819
Independent reflections	2964 [R(int)= 0.0943]
Final R indices [I>2sigma(I)]	R1= 0.0513, wR2= 0.1112
R indices (all data)	R1= 0.1077, wR2= 0.1322
Goodness−of−fit on F2	1.016
Highest difference peak and deepest hole (e Å^{-3})	0.288 and -0.264

Supplementary Table 6 Bond lengths (Å) and angles for CH_3-HO-PBA

O2−B1	1.379(2)	C11−C10−C9	122.45(18)
O2−B2	1.397(3)	C11−C10−H10	118.8
O1−C8	1.387(2)	C8−C14−H14	120.7
O1−B1	1.378(3)	C8−C14−C13	118.59(18)
O3−H3	0.8400	C13−C14−H14	120.7
O3−C2	1.382(2)	C10−C11−C13	118.06(18)
O4−H4	0.8400	C10−C11−C12	121.44(18)
O4−B2	1.342(2)	C13−C11−C12	120.49(18)
C9−C8	1.391(2)	O3−C2−C1	121.77(17)
C9−C10	1.405(3)	O3−C2−C3	116.75(18)
C9−B2	1.549(3)	C3−C2−C1	121.47(18)
C8−C14	1.384(3)	C5−C6−H6	118.4
C10−H10	0.9500	C5−C6−C1	123.29(19)
C10−C11	1.385(3)	C1−C6−H6	118.4
C14−H14	0.9500	C6−C5−C4	117.64(18)
C14−C13	1.385(3)	C6−C5−C7	120.41(18)
C11−C13	1.392(3)	C4−C5−C7	121.94(18)
C11−C12	1.507(3)	C2−C1−C6	116.75(17)
C2−C1	1.397(3)	C2−C1−B1	123.14(17)
C2−C3	1.384(3)	C6−C1−B1	120.11(18)
C6−H6	0.9500	C14−C13−C11	121.58(18)
C6−C5	1.387(3)	C14−C13−H13	119.2
C6−C1	1.399(3)	C11−C13−H13	119.2
C5−C4	1.389(3)	C2−C3−H3A	120.1
C5−C7	1.509(3)	C2−C3−C4	119.71(19)
C1−B1	1.545(3)	C4−C3−H3A	120.1
C13−H13	0.9500	C5−C4−H4A	119.4
C3−H3A	0.9500	C3−C4−C5	121.14(18)

C3−C4	1.386(3)	C3−C4−H4A	119.4
C4−H4A	0.9500	C11−C12−H12A	109.5
C12−H12A	0.9800	C11−C12−H12B	109.5
C12−H12B	0.9800	C11−C12−H12C	109.5
C12−H12C	0.9800	H12A−C12−H12B	109.5
C7−H7A	0.9800	H12A−C12−H12C	109.5
C7−H7B	0.9800	H12B−C12−H12C	109.5
C7−H7C	0.9800	C5−C7−H7A	109.5
B1−O2−B2	121.88(16)	C5−C7−H7B	109.5
B1−O1−C8	122.00(15)	C5−C7−H7C	109.5
C2−O3−H3	109.5	H7A−C7−H7B	109.5
B2−O4−H4	109.5	H7A−C7−H7C	109.5
C8−C9−C10	116.81(18)	H7B−C7−H7C	109.5
C8−C9−B2	118.01(17)	O2−B1−C1	120.84(18)
C10−C9−B2	125.14(17)	O1−B1−O2	121.07(18)
O1−C8−C9	120.54(17)	O1−B1−C1	118.09(17)
C14−C8−O1	116.96(16)	O2−B2−C9	116.48(17)
C14−C8−C9	122.50(18)	O4−B2−O2	120.83(18)
C9−C10−H10	118.8	O4−B2−C9	122.69(18)

Supplementary Table 7 Hydrogen bond data for CH_3-HO-PBA

D−H...A	d(D−H)	d(H...A)	d(D...A)	<(DHA)
O3−H3...O2	0.8401 (14)	1.9743 (14)	2.7307 (20)	149.334 (103)
O4-H4...O3^{i}	0.8399 (14)	1.9635 (14)	2.7942 (20)	169.816 (103)
C14-H14...O1^{ii}	0.9501 (18)	2.8202 (12)	3.5042 (22)	129.697 (112)

Symmetry codes: (i) *x*, 1+*y*, *z*; (ii) 1−*x*, 1-*y*, 1−*z*.

Supplementary Table 8 Crystal data and structure refinements for CF_3-HO-PBA

Empirical formula	$C_{14}H_8B_2F_6O_4$
Formula weight	375.82
Temperature (K)	120
Crystal size (mm^3)	0.15 x 0.1 x 0.1
Crystal system	monoclinic
Space group	P 1 21/c 1
a (Å)	15.995(3)
b (Å)	4.9175(9)
c (Å)	18.567(3)
α (°)	90
β (°)	92.217(7)
γ (°)	90
V (Å^3)	1459.3(4)
Z	4
Density (g/cm^3)	1.711
F (000)	752
Theta range for data collection	2.50 to 27.42
Index ranges	−20<=h<=14, −5<=k<=6, −24<=l<=24
Reflections collected	11314
Independent reflections	3297 [R(int)= 0.0399]
Final R indices [I>2sigma(I)]	R1= 0.0615, wR2= 0.1545
R indices (all data)	R1= 0.0722, wR2= 0.1610
Goodness−of−fit on F^2	1.102
Highest difference peak and deepest hole (e Å^{-3})	0.338 and -0.359

Supplementary Table 9 Bond lengths (Å) and angles for CF_3-HO-PBA

F4−C14	1.338(3)	C12−C11−C14	118.9(2)
F5−C14	1.338(3)	C10−C11−C14	120.0(2)
F6−C14	1.344(3)	C6−C1−C2	118.2(2)
O2−B1	1.401(3)	C6−C1−B1	117.6(2)
O2−B2	1.376(3)	C2−C1−B1	124.2(2)
O1−H1	0.8400	O3−C6−C1	121.1(2)
O1−B1	1.336(4)	O3−C6−C5	116.9(2)
O4−H4	0.8400	C5−C6−C1	122.0(2)
O4−C13	1.369(3)	C10−C9−H9	118.8
F1−C7	1.331(3)	C10−C9−C8	122.4(2)
O3−C6	1.384(3)	C8−C9−H9	118.8
O3−B2	1.371(3)	C11−C10−H10	120.5
F3−C7	1.336(4)	C9−C10−C11	119.0(2)
F2−C7	1.327(4)	C9−C10−H10	120.5
C12−H12	0.9500	C1−C2−H2	119.4
C12−C13	1.393(4)	C3−C2−C1	121.1(3)
C12−C11	1.389(4)	C3−C2−H2	119.4
C13−C8	1.415(3)	C2−C3−H3	120.5
C11−C10	1.393(4)	C2−C3−C4	118.9(3)
C11−C14	1.502(4)	C4−C3−H3	120.5
C1−C6	1.397(4)	C6−C5−H5	121
C1−C2	1.401(4)	C6−C5−C4	118.0(3)
C1−B1	1.561(4)	C4−C5−H5	121
C6−C5	1.390(4)	C3−C4−C7	120.2(3)
C9−H9	0.9500	C5−C4−C3	121.8(2)
C9−C10	1.381(4)	C5−C4−C7	118.1(3)
C9−C8	1.401(4)	C13−C8−B2	121.9(2)
C10−H10	0.9500	C9−C8−C13	116.9(2)

C2−H2	0.9500	C9−C8−B2	121.2(2)
C2−C3	1.386(4)	F4−C14−F6	106.5(2)
C3−H3	0.9500	F4−C14−C11	112.5(2)
C3−C4	1.392(4)	F5−C14−F4	106.8(2)
C5−H5	0.9500	F5−C14−F6	106.8(2)
C5−C4	1.391(4)	F5−C14−C11	112.5(2)
C4−C7	1.508(4)	F6−C14−C11	111.4(2)
C8−B2	1.558(4)	F1−C7−F3	105.9(3)
B2−O2−B1	122.0(2)	F1−C7−C4	112.9(2)
B1−O1−H1	109.5	F3−C7−C4	112.0(3)
C13−O4−H4	109.5	F2−C7−F1	106.5(3)
B2−O3−C6	121.5(2)	F2−C7−F3	107.2(3)
C13−C12−H12	120.5	F2−C7−C4	111.8(3)
C11−C12−H12	120.5	O2−B1−C1	115.8(2)
C11−C12−C13	119.1(2)	O1−B1−O2	121.4(2)
O4−C13−C12	115.9(2)	O1−B1−C1	122.7(3)
O4−C13−C8	122.6(2)	O2−B2−C8	119.2(2)
C12−C13−C8	121.5(2)	O3−B2−O2	121.9(2)
C12−C11−C10	121.0(2)	O3−B2−C8	118.9(2)

Supplementary Table 10 Hydrogen bond data for CF_3-HO-PBA

D−H...A	d(D−H)	d(H...A)	d(D...A)	<(DHA)
O4−H4...O2	0.8398 (19)	1.9338 (18)	2.6896 (19)	149.177 (139)
C9-H9...F3^{i}	0.9500 (26)	2.6448 (22)	3.3071 (34)	127.221 (162)

Symmetry codes: (i) 1−*x*, -0.5+*y*, 0.5−*z*.

Supplementary Table 11 Equilibrium constants for the transformation from HO-PBA dimer to HO-PBA trimer–D_2O complex, calculated at different n values and D_2O amounts under room temperature

n	D_2O (μL)				
	2	4	6	8	10
1	13.2	44.6	104.9	208.5	368.3
2	2063.1	2036.5	2023.6	2041.1	2076.8
3	6.9×10^5	2.4×10^5	9.5×10^4	4.3×10^4	2.2×10^4
4	6.4×10^8	1.1×10^8	1.6×10^7	2.5×10^6	5.6×10^5

Supplementary references

1. Tzoumani, I., Soto Beobide, A., Iatridi, Z., Voyiatzis, G. A., Bokias, G. & Kallitsis, J. K. Glycidyl methacrylate-based copolymers as healing agents of waterborne polyurethanes. *Int. J. Mol. Sci.* **23**, 8118 (2022).
2. Zhang, G., Zhang, Q., Cheng, T., Zhan, X. & Chen, F. Polyols-infused slippery surfaces based on magnetic Fe_3O_4-functionalized polymer hybrids for enhanced multifunctional anti-icing and deicing properties. *Langmuir* **34**, 4052-4058 (2018).
3. Yang, W. J., Pranantyo, D., Neoh, K.-G., Kang, E.-T., Teo, S. L.-M. & Rittschof, D. Layer-by-layer click deposition of functional polymer coatings for combating marine biofouling. *Biomacromolecules* **13**, 2769-2780 (2012).
4. Xia, J., Lu, D., Han, Y., Wang, J., Hong, Y., Zhao, P., Fang, Q. & Lin, Q. Facile multifunctional IOL surface modification via poly(PEGMA-co-GMA) grafting for posterior capsular opacification inhibition. *RSC Adv.* **11**, 9840-9848 (2021).
