# Supplementary figures and images for: Water-stable boroxine structure with dynamic covalent bonds
Source: Nat Commun. 2024 Feb 8;15:1207. doi: 10.1038/s41467-024-45464-z (PMC10853236; doi:10.1038/s41467-024-45464-z)

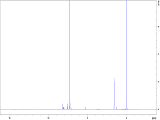

Supplement: Supplementary file 3 — Source data [file 41467_2024_45464_MOESM3_ESM.zip › source data/CF3-HO-PBA/CF3-HO-PBA dimer NMR/H/pdata/1/thumb.png]

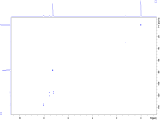

Supplement: Supplementary file 3 — Source data [file 41467_2024_45464_MOESM3_ESM.zip › source data/CF3-HO-PBA/CF3-HO-PBA dimer NMR/HSQC/pdata/1/thumb.png]

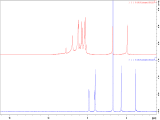

Supplement: Supplementary file 3 — Source data [file 41467_2024_45464_MOESM3_ESM.zip › source data/CF3-HO-PBA/CF3-HO-PBA trimer NMR/1/pdata/1/thumb.png]

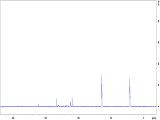

Supplement: Supplementary file 3 — Source data [file 41467_2024_45464_MOESM3_ESM.zip › source data/CF3-HO-PBA/CF3-HO-PBA trimer NMR/2/pdata/1/thumb.png]

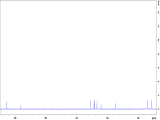

Supplement: Supplementary file 3 — Source data [file 41467_2024_45464_MOESM3_ESM.zip › source data/CH3-HO-PBA/CH3-HO-PBA dimer NMR/C/pdata/1/thumb.png]

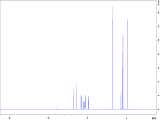

Supplement: Supplementary file 3 — Source data [file 41467_2024_45464_MOESM3_ESM.zip › source data/CH3-HO-PBA/CH3-HO-PBA dimer NMR/H/pdata/1/thumb.png]

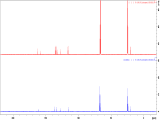

Supplement: Supplementary file 3 — Source data [file 41467_2024_45464_MOESM3_ESM.zip › source data/CH3-HO-PBA/CH3-HO-PBA trimer NMR/C/pdata/1/thumb.png]

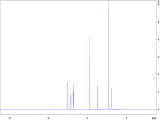

Supplement: Supplementary file 3 — Source data [file 41467_2024_45464_MOESM3_ESM.zip › source data/CH3-HO-PBA/CH3-HO-PBA trimer NMR/H/pdata/1/thumb.png]

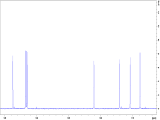

Supplement: Supplementary file 3 — Source data [file 41467_2024_45464_MOESM3_ESM.zip › source data/HO-PBA/HO-PBA dimer NMR/C/pdata/1/thumb.png]
